# Isolation and Characterization of 5-(1-Hydroxyethyl)-Dihydro-2-Furanone from *Angiopteris evecta* with Potent Anti-Inflammatory and Anti-Leukemic Activities

**DOI:** 10.3390/ijms27031399

**Published:** 2026-01-30

**Authors:** Lapamas Rueankham, Natsima Viriyaadhammaa, Wenxian Yin, Yuanzhi Liu, Sawitree Chiampanichayakul, Methee Rungrojsakul, Trinnakorn Katekunlaphan, Siriporn Okonogi, Aroonchai Saiai, Arihiro Iwasaki, Christian Nanga Chick, Toyonobu Usuki, Songyot Anuchapreeda

**Affiliations:** 1 Department of Medical Technology, Faculty of Associated Medical Sciences, Chiang Mai University, Chiang Mai 50200, Thailand; lapamas_ru@cmu.ac.th (L.R.); natsima.v@cmu.ac.th (N.V.); yinwenxian@swmu.edu.cn (W.Y.); sawitree.chiampa@cmu.ac.th (S.C.); 2 Cancer Research Unit of Associated Medical Sciences (AMS CRU), Faculty of Associated Medical Sciences, Chiang Mai University, Chiang Mai 50200, Thailand; 3 Department of Pharmacy, The Affiliated Hospital, Southwest Medical University, Luzhou 646000, China; liuyuanzhi121@swmu.edu.cn; 4 Center of Excellence in Pharmaceutical Nanotechnology, Faculty of Pharmacy, Chiang Mai University, Chiang Mai 50200, Thailand; okng2000@gmail.com; 5 Department of Traditional Chinese Medicine, Faculty of Science, Chandrakasem Rajabhat University, Bangkok 10900, Thailand; methee.r@chandra.ac.th; 6 Department of Chemistry, Faculty of Science, Chandrakasem Rajabhat University, Bangkok 10900, Thailand; trinnakorn.k@chandra.ac.th; 7 Department of Pharmaceutical Sciences, Faculty of Pharmacy, Chiang Mai University, Chiang Mai 50200, Thailand; 8 Department of Chemistry, Faculty of Science, Chiang Mai University, Chiang Mai 50200, Thailand; aroonchai.s@cmu.ac.th; 9 Department of Applied Chemistry, Faculty of Science and Engineering, Chuo University, Tokyo 112-8551, Japan; aiwasaki686@g.chuo-u.ac.jp; 10 Department of Materials and Life Sciences, Faculty of Science and Technology, Sophia University, Tokyo 102-8554, Japan; chicknanga0@gmail.com

**Keywords:** acute myeloid leukemia, *Angiopteris evecta*, furanone derivatives, ternary mixture, WT1, apoptosis, cell cycle arrest, network pharmacology, molecular docking, anti-inflammatory activity, MAPK signaling

## Abstract

Acute myeloid leukemia (AML) is a heterogeneous hematological malignancy with poor prognosis, frequent relapse, and treatment-related toxicity. The discovery of novel anti-leukemic agents with improved selectivity remains an urgent clinical need. In this study, rhizomes of *Angiopteris evecta*, a medicinal plant used in Thai traditional medicine, were collected from twelve locations in Thailand and extracted using solvents of increasing polarity. Among thirty-six crude fractional extracts, the ethyl acetate crude fractional extract from source No. 003 (AE EtOAc No. 003) exhibited the strongest cytotoxic activity against KG-1a and EoL-1 leukemic cell lines, with low toxicity toward normal peripheral blood mononuclear cells. Bioactivity-guided fractionation yielded the ternary mixture, a furanone-rich mixture dominated by 5-(1-hydroxyethyl)-dihydro-2-furanone. The ternary mixture inhibited leukemic cell proliferation by inducing apoptosis, causing cell cycle arrest, and downregulating WT1 expression in EoL-1 cells. Network pharmacology and molecular docking analyses implicated AKT1, MAPK signaling, apoptosis-related pathways, and WT1 as key molecular targets. In addition, AE EtOAc No. 003 and the ternary mixture suppressed TNF-α and IL-6 production in LPS-stimulated macrophages. Collectively, *A. evecta*-derived furanone compounds represent promising lead candidates for anti-leukemic drug development.

## 1. Introduction

Acute myeloid leukemia (AML) is the subtype of leukemia with an increasing prevalence and persistently low survival rates, accompanied by a yearly rise in mortality [1]. AML is characterized by the accumulation of undifferentiated blast cells and disruption of the number and function of normal blood cells, resulting from abnormal proliferation and impaired differentiation of hematopoietic stem and progenitor cells (HSPCs) [2,3]. Genetic alterations, including chromosomal aberrations and single-nucleotide variants (SNVs), can transform hematopoietic stem cells or progenitor cells into leukemic stem cells (LSCs) through mutations in genes such as *DNMT3A*, *FLT3*, *NPM1*, *CEBPA*, or *WT1* [4,5]. LSCs possess self-renewal capacity, remain dormant in the G0 phase, and lack differentiation potential, enabling them to evade chemotherapeutic eradication and contribute to AML relapse [6]. *Wilms’* *tumor 1* (*WT1*) gene encodes a zinc finger transcription factor (WT1 protein) that was originally defined as a tumor suppressor gene and plays critical roles in development, differentiation arrest, apoptosis, and proliferation during normal hematopoiesis and urogenital development. Under physiological conditions, WT1 protein is expressed at low or undetectable levels. However, elevated WT1 expression has been implicated in the progression of various solid cancers and leukemogenesis, suggesting that *WT1* gene can also function as an oncogene [7]. The *WT1* gene is frequently mutated or overexpressed in AML, contributing to disease progression and poor prognosis [8]. Overexpression of WT1 protein enhances leukemic blast proliferation, inhibits apoptosis, and promotes cell survival. Consequently, WT1 protein serves a biomarker for aggressive AML and a predictor of disease relapse [9].

Although intensive chemotherapy, particularly the “3 + 7” regimen, has been widely used for the treatment of AML for several decades, chemotherapeutic drugs primarily target rapidly dividing cells and therefore also affect normal proliferating cells. This lack of selectivity results in significant adverse effects, including nausea, vomiting, gastrointestinal toxicity, mucositis, and alopecia [10,11]. Moreover, the long-term survival rate of AML patients remains below 40% following initial treatment. Approximately 20% of AML patients who achieve complete remission after intensive chemotherapy subsequently relapse, often with incomplete hematologic recovery and drug resistance [12,13]. To overcome these limitations, complementary and alternative medicine (CAM), particularly medicinal plants, has gained increasing interest as a source of novel therapeutic agents with higher efficacy and lower toxicity. Herbal medicines have been used for disease treatment for centuries, and numerous medicinal plants have been recommended for leukemia therapy. For example, vincristine sulfate (Oncovin^®^) and vinblastine sulfate (Velban^®^), isolated from *Catharanthus roseus*, are clinically used anticancer agents, including for leukemia treatment, through their ability to disrupt microtubule dynamics [14]. Flavopiridol, an alkaloid isolated from the bark of *Dysoxylum binetariferum*, exhibits broad spectrum antileukemic activity by inhibiting cyclin-dependent kinases (CDKs), inducing apoptosis, promotion oxidative stress, and exerting anti-angiogenic effects. Flavopiridol is currently used in combination therapy with cytotoxic agents such as cytarabine and mitoxantrone, a regimen known as alvocidib [15,16].

*Angiopteris evecta* is commonly known as giant fern or king fern and locally referred to as “Wan Keab Rad” in Thailand, belonging to the family Marattiaceae. This species is widely distributed in India, and is also found in Malaysia, Indonesia, Thailand, Australia, and New Guinea [17]. In Thai traditional medicine, *A. evecta* rhizome is an ingredient of the remedy “Kheaw-Hom” and is traditionally used as a diuretic, antipyretic, analgesic, and anti-diarrheal agent [18,19]. Previous studies have demonstrated that methanolic extracts of *A. evecta* leaves possess various bioactivities, including antioxidant, anti-inflammatory, anti-tuberculosis, anti-bacterial, and anti-fungal activity [20,21,22]. However, studies investigating the anticancer activity of *A. evecta* remain limited. In 2022, Sara S.C. et al. reported that the ethanolic extract of *A. evecta* exhibited significant antiproliferative activity against malignant colon cancer cells (HT-29) by inducing apoptosis and causing cell-cycle arrest at the G0/G1 phase [23]. Phytochemical investigations of other *Angiopteris* species have been identified compounds such as angiopteroside, lactones, coumarin, β-sitosterol, and furans [24,25], which exhibit diverse biological activities, including anti-bacterial, anti-inflammatory, and anti-adipogenic activity [26,27]. Angiopteroside, isolated from *A. evecta* rhizome has also demonstrated inhibitory activity against HIV-1 reverse transcriptase [28]. Nevertheless, the antileukemic effects of *A. evecta* extracts and their active constituents have not yet been elucidated. In this present study, dried rhizomes of *A. evecta* were collected from 12 different locations in Thailand and extracted using *n*-Hexane (*n*-Hex), ethyl acetate (EtOAc), and ethanol (EtOH). These extracts were screened and compared for cytotoxic effects against leukemic cell lines (KG-1a and EoL-1 cells). The most effective crude fractional extract was further subjected to purification and identification of the active compounds, followed by in vitro evaluation of their antileukemic activities.

## 2. Results

### 2.1. Yield of Extracts

The crude fractional extracts were prepared from dried rhizomes of *A. evecta* collected from 12 different locations in Thailand using *n*-Hex, EtOAc, and EtOH as extraction solvents. As summarized in Table 1, the EtOH extracts consistently yielded the highest extraction efficiency across all 12 samples, followed by the EtOAc extracts, whereas the *n*-Hex extracts produced the lowest yields.

### 2.2. Cytotoxic Effects of A. evecta Crude Functional Extracts on KG-1a and EoL-1 Leukemic Cells and PBMCs

A total of 36 Crude fractional extracts of *A. evecta* were screened for cytotoxic activity against leukemic cell lines, including KG-1a and EoL-1, using the MTT assay. The EtOH crude fractional extracts showed no cytotoxicity toward either KG-1a or EoL-1 cells, with the IC_50_ values greater than 100 µg/mL, as summarized in Table 2. Similarly, the *n*-Hex crude fractional extracts exhibited no cytotoxic effects on KG-1a cells. However, several *n*-Hex extracts including No. 001 (from Bangkok), 006 (from Phitsanulok), 007 (from Lopburi), 008 (from Phuket), 009 (from Chiang Mai 1), 010 (from Chiang Mai 2), and 012 (from Tak) demonstrated moderate cytotoxicity against EoL-1 cells, with IC_50_ values ranging from 70.68 ± 5.25 to 98.97 ± 12.78 µg/mL. In contrast, most EtOAc crude fractional extracts exhibited pronounced cytotoxic effects on both KG-1a and, in particular, EoL-1 cells. Notably, among all active EtOAc extracts, the EtOAc crude fractional extract No. 003 (AE EtOAc No. 003; from Bangkok 3) showed the strongest cytotoxic activity against both cell lines, with IC_50_ values of 23.29 ± 3.78 for KG-1a cells and 13.29 ± 1.90 µg/mL for EoL-1 cells (Table 2). Furthermore, AE EtOAc No. 003 exhibited low toxicity toward normal peripheral blood mononuclear cells (PBMCs) at low concentrations (3.125–25 µg/mL). Cytotoxic effects were observed only at higher concentrations, with an IC_50_ value of 40.91 ± 6.17 µg/mL. Based on its potent antileukemic activity and comparatively low toxicity toward normal cells, AE EtOAc No. 003 was selected as the candidate crude fractional extract of *A. evecta* for further investigation.

### 2.3. Purification and Cytotoxic Screening of Active Compounds from A. evecta Against KG-1a and EoL-1 Leukemic Cell, and Peripheral Blood Mononuclear Cells (PBMCs)

**Figure 1:**
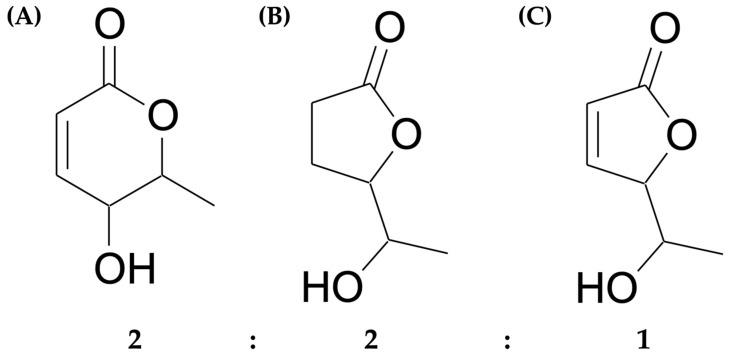
Chemical structures of three compounds comprising in the ternary mixture, isolated from AE EtOAc No. 003: (**A**) *epi*-osmundalactone (5,6-dihydro-5-hydroxy-6-methyl-2H-pyran-2-one), (**B**) 5-(1-hydroxyethyl)-dihydro-2-furanone, and (**C**) 5-(1-hydroxyethyl)-2(5H)-furanone, in the ratio of 2:2:1, respectively.

Based on the pronounced cytotoxic activity of AE EtOAc No. 003 against both KG-1a and EoL-1 cells, AE EtOAc No. 003 (3 g) was subjected to compound separation and purification using column chromatography. Four purified compounds were obtained and subsequently screened for cytotoxicity against KG-1a and EoL-1 cells, as well as normal PBMCs, using the MTT assay. As summarized in Supplementary Table S1. Among the purified compounds, purified compound 4, obtained as a light-yellow viscous oil, exhibited the highest yield (30.46%) and the most potent cytotoxic activity against both KG-1a and EoL-1 leukemic cells (with IC_50_ values of 9.73 ± 0.24 and 8.10 ± 0.73 µg/mL, respectively), while maintaining lower toxicity toward normal PBMCs. Consistent with these findings, the selectivity index (SI) of purified compound 4 indicated a higher efficacy against leukemic cells compared with normal PBMCs, with the higher SI values than the SI value of the anticancer agent (doxorubicin) for KG-1a cells. Nevertheless, the SI values for both purified compound 4 and doxorubicin were greater than 1, indicating the greater specific performance against the leukemic cells than normal PBMCs (Table 3). To determine whether purified compound 4 was chemically homogenous, further purification was performed using high-performance liquid chromatography (HPLC), followed by structural characterization using multiple orthogonal analytical techniques, including infrared (IR) spectroscopy, Gas chromatography-Mass spectrometry (GC–MS), and nuclear magnetic resonance (NMR) spectroscopy (^1^H, ^13^C, COSY, HMBC). The detailed spectral data are provided in Supplementary Data S1, Tables S2–S4, and Figures S1–S4. The NMR spectra compared with previously reported data by Chen Y., 2010 [24] showed a single peak on the HPLC chromatogram, however, it was revealed a mixture of three coeluting chemical compounds (as the ternary mixture) in a 2:2:1 ratio: *epi*-osmundalactone (5,6-dihydro-5-hydroxy-6-methyl-2H-pyran-2-one), 5-(1-hydroxyethyl)-dihydro-2-furanone, and 5-(1-hydroxyethyl)-2(5H)-furanone (Figure 1). Based on its superior cytotoxic activity and selectivity toward leukemic cells, the ternary mixture was selected for further biological and mechanistic investigation.

### 2.4. Effects of AE EtOAc No. 003 and the Ternary Mixture on the Proliferation Rate of KG-1a and EoL-1 Leukemic Cells

**Figure 2:**
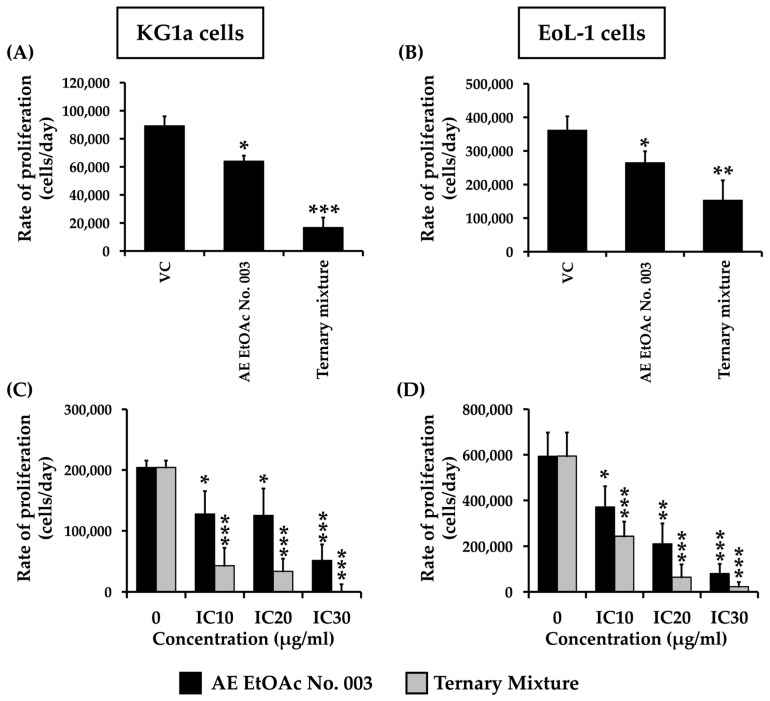
Proliferation rate of leukemic cells following treatment with AE EtOAc No. 003 and the ternary mixture. (**A**) KG-1a and (**B**) EoL-1 cells were treated with AE EtOAc No. 010 and the ternary mixture at IC_20_ concentrations for 12, 24, and 48 h. (**C**) KG-1a and (**D**) EoL-1 cells were treated with varying concentrations (IC_10_, IC_20_, and IC_30_) of AE EtOAc No. 003 and the ternary mixture for 48 h. Total cell numbers were determined using the trypan blue exclusion assay. Data are presented as the mean ± SD of three independent experiments. Asterisks (*) indicate statistically significant differences compared with the vehicle control (* *p* < 0.05, ** *p* < 0.01, and *** *p* < 0.001).

To determine whether the cytotoxic activity of the ternary mixture translated into functional inhibition of leukemic cell growth, its effects on cell proliferation were further evaluated. KG-1a and EoL-1 cells were treated with AE EtOAc No. 003 and the ternary mixture under various experimental conditions. Cell viability was subsequently assessed using the trypan blue exclusion assay. As shown in Figure 2, the proliferation rates of both KG-1a and EoL-1 cells decreased following treatment with increasing concentrations of AE EtOAc No. 003 and the ternary mixture, as well as with prolonged incubation times. These results indicate that both AE EtOAc No. 003 and the ternary mixture effectively inhibited leukemic cell proliferation in a dose- and time-dependent manner.

### 2.5. Effects of AE EtOAc No. 003 and the Ternary Mixture on WT1 Expression in Leukemic Cells

**Figure 3:**
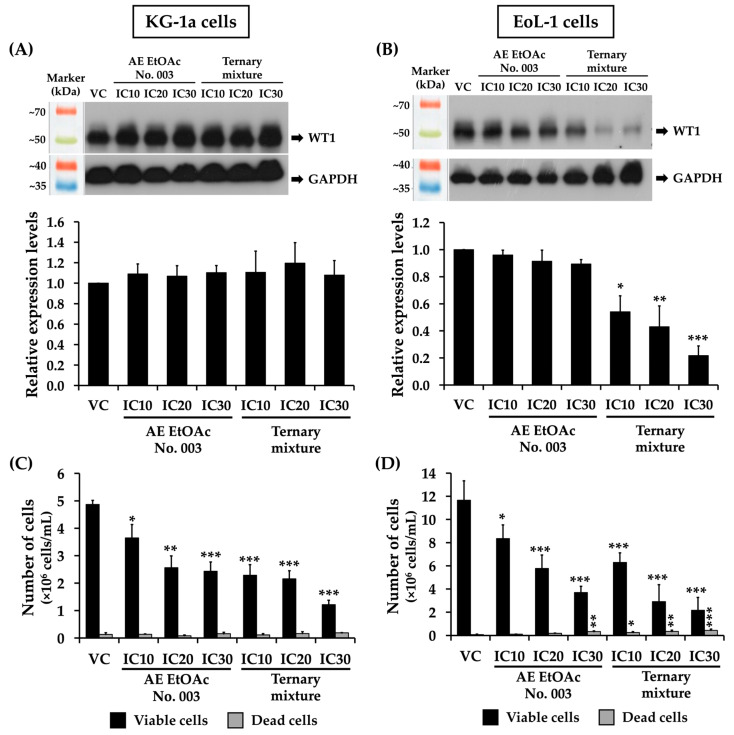
Effects of AE EtOAc No. 003 and the ternary mixture on WT1 expression in KG-1a and EoL-1 cells. WT1 protein expression in (**A**) KG-1a and (**B**) EoL-1 cells following treatment with various concentrations of AE EtOAc No. 003 or the ternary mixture for 48 h was assessed using Western blotting and quantified by scanning densitometric analysis. WT1 protein levels were normalized to GAPDH. The total cell numbers of (**C**) KG-1a and (**D**) EoL-1 cells following treatment with AE EtOAc No. 003 or the ternary mixture for 48 h were determined using the trypan blue exclusion assay. Data are presented as mean ± SD of three independent experiments. Asterisks (*) indicate statistically significant differences compared with the vehicle control (* *p* < 0.05, ** *p* < 0.01, and *** *p* < 0.001).

Overexpression of WT1 has been associated with poor prognosis and disease progression in AML [7,29]. Reduction in WT1 overexpression in AML patients has been reported to decrease relapse rates, improve long-term prognosis, and increase overall survival (OS) [30,31]. In the present study, the effects of the *A. evecta* crude fractional extract and its active compound on WT1 expression in leukemic cells were further investigated. KG-1a and EoL-1 cells were treated with various concentrations of AE EtOAc No. 003 or the ternary mixture for 48 h, after which WT1 protein expression was analyzed by Western blotting. The results demonstrated that neither AE EtOAc No. 003 nor the ternary mixture significantly affected WT1 expression in KG-1a cells (Figure 3A), despite a reduction in the total number of KG-1a cells following treatment (Figure 3C). In contrast, WT1 expression in EoL-1 cells was slightly reduced following treatment with AE EtOAc No. 003. Notably, treatment with the ternary mixture resulted in a significant downregulation of WT1 in EoL-1 cells (Figure 3B), which correlated with a concentration-dependent decrease in EoL-1 cell numbers (Figure 3D). These findings suggest that the suppression of EoL-1 cell proliferation may be partially attributable to the inhibitory effects of AE EtOAc No. 003 and, in particular, the ternary mixture on WT1 expression.

### 2.6. Molecular Docking of Active Compounds with AML-Related Targets

**Figure 4:**
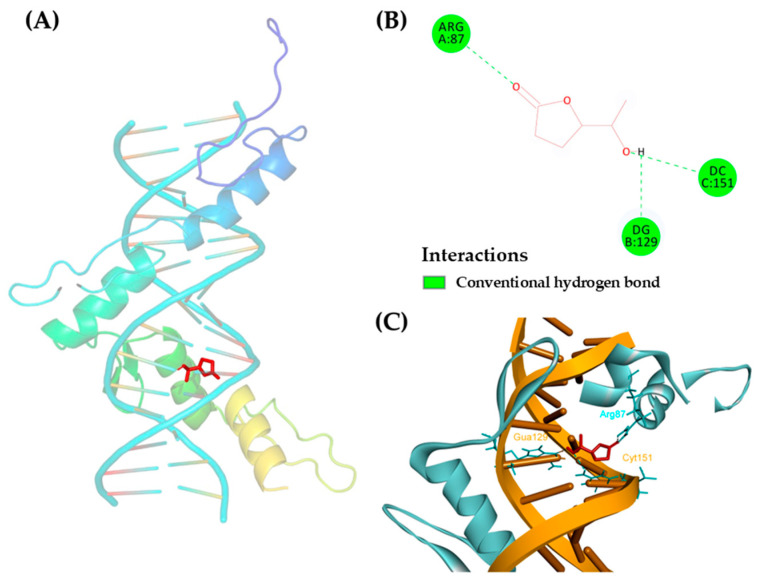
Molecular docking results of WT1 and 5-(1-hydroxyethyl)-dihydro-2-furanone. (**A**) Overall 3D structure of the ligand-protein-DNA complex. (**B**) 2D schematic of ligand interactions, showing conventional hydrogen bonds with Arg87, DG129, and DC151. (**C**) 3D visualization of the binding pocket at the interface of the WT1 protein (cyan) and DNA (orange). The ligand is highlighted as red sticks.

To investigate the molecular interaction between the three compounds comprised in the ternary mixture and AML-associated proteins, a docking-based analysis was performed. The crystal structure of WT1 (PDB ID: 4R2E) and key kinases (ERK1, ERK2, etc.) were selected as docking receptors, with ligand structures obtained from the PubChem database. Based on the docking scores, 5-(1-hydroxyethyl)-dihydro-2-furanone exhibited the strongest binding affinity toward WT1 (−6.3 kcal/mol). However, 5-(1-hydroxyethyl)-2(5H)-furanone and *epi*-osmundalactone yielded scores of −6.2 kcal/mol and −5.3 kcal/mol, respectively (Table 4). In addition, all three compounds showed significant affinities with ERK1 and ERK2 (all ≥ −5.6 kcal/mol), suggesting a potential multi-target inhibitory effect on the MAPK pathway. Furthermore, 5-(1-hydroxyethyl)-dihydro-2-furanone established strong conventional hydrogen bonding interactions with three key sites at the protein-DNA interface: amino acid residue ARG87 (Chain A) and nucleotides DG129 (Chain B) and DC151 (Chain C) (Figure 4A–C). These results indicate that the ternary mixture stabilized within the WT1 binding pocket, potentially interfering with its biological activity in AML cells.

### 2.7. Targets Prediction and Screening Using Network Pharmacology

#### 2.7.1. Prediction of Anti-Acute Myeloid Leukemia (AML) Targets of 5-(1-Hydroxyethyl)-Dihydro-2-Furanone, the Active Compound from *A. evecta*

**Figure 5:**
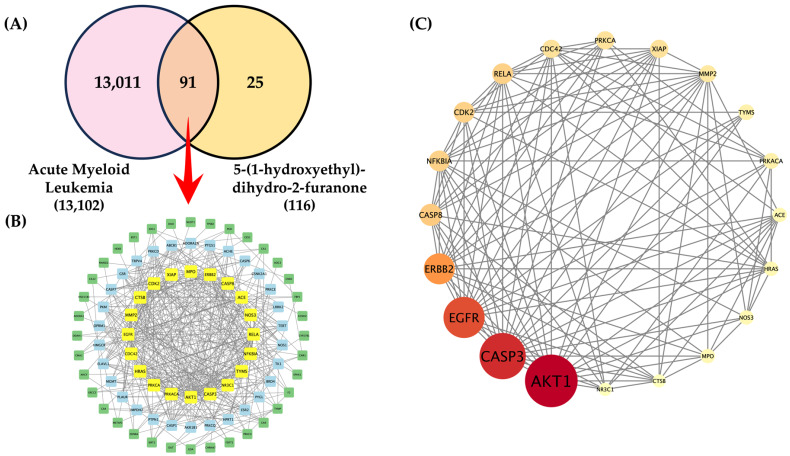
Network pharmacology analysis of the anti-AML activity of 5-(1-hydroxyethyl)-dihydro-2-furanone. (**A**) Venn diagram showing the intersection between AML-associated proteins and the predicted target proteins of 5-(1-hydroxyethyl)-dihydro-2-furanone. (**B**) Protein-protein interaction (PPI) network of the 91 overlapping target proteins associated with both AML and 5-(1-hydroxyethyl)-dihydro-2-furanone. (**C**) Top 20 hub target proteins involved in the predicted anti-AML activity of 5-(1-hydroxyethyl)-dihydro-2-furanone.

According to the compound-WT1 protein molecular docking, 5-(1-hydroxyethyl)-dihydro-2-furanone was further predicted as a potential target protein involved in the anti-AML activity using network pharmacology analysis. A total of 13,102 AML-associated target proteins were retrieved from the DisGeNET and GeneCards databases. In parallel, 116 potential target proteins of 5-(1-hydroxyethyl)-dihydro-2-furanone were collected from SwissTargetPrediction and PharmMapper online database. After removal of duplicate entries using a Venn diagram, 91 common target proteins were identified as overlapping targets between AML-related proteins and those associated with 5-(1-hydroxyethyl)-dihydro-2-furanone (Figure 5A). To further elucidate the potential mechanisms by which these 91 target proteins mediate the effects of 5-(1-hydroxyethyl)-dihydro-2-furanone on AML cell growth, a protein-protein interaction (PPI) network was constructed using the STRING database (Figure 5B). Degree values of the network nodes were calculated and analyzed using Cytoscape to rank the target proteins according to their connectivity. The top 20 hub proteins, comprising 150 interaction edges, were visualized as shown in Figure 5C. Among these, AKT1 exhibited the highest degree values and was predicted to be a key target potentially activated or suppressed by 5-(1-hydroxyethyl)-dihydro-2-furanone, thereby contributing to the regulation of leukemic cell proliferation. In addition, several other hub proteins associated with leukemic cell growth and apoptosis, including caspase-3, EGFR, ERBB2, and caspase-8, were also identified within the PPI network.

#### 2.7.2. Gene Ontology (GO) and KEGG Analysis

**Figure 6:**
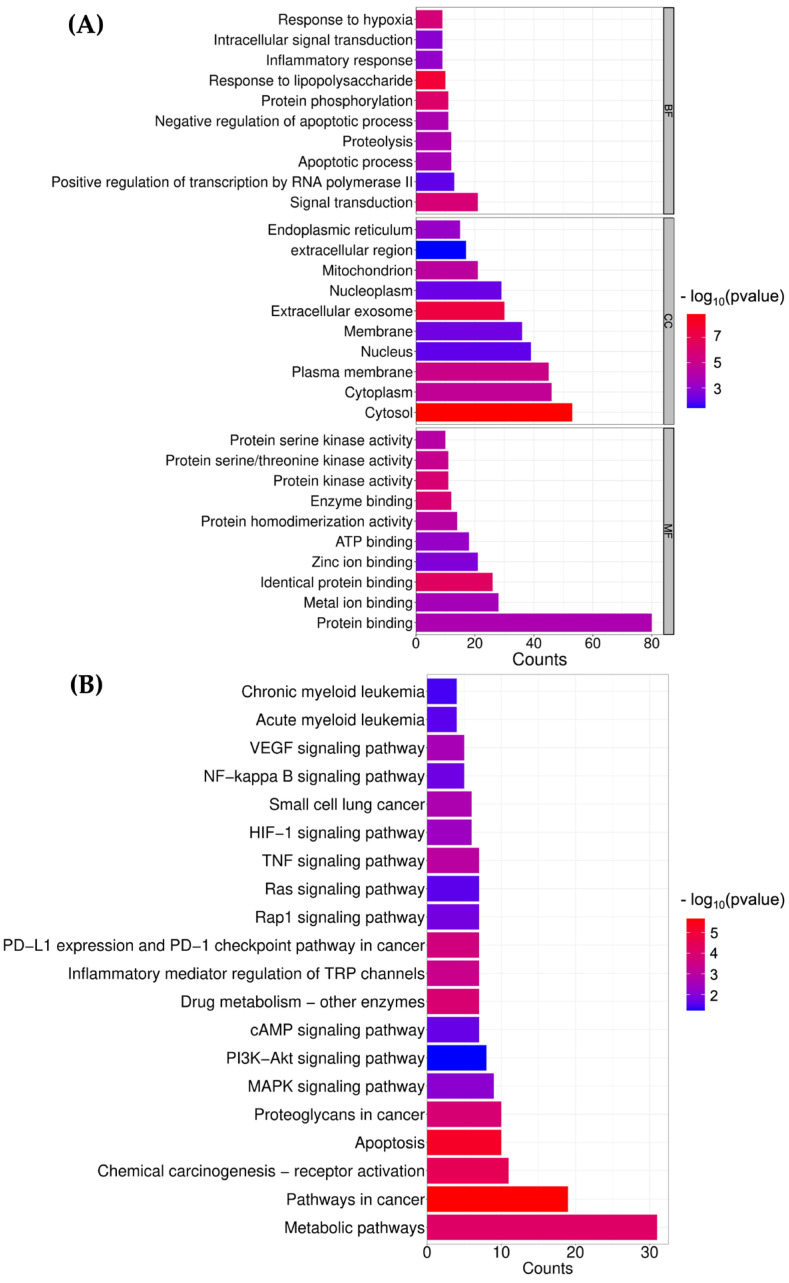
GO and KEGG enrichment analyses of target proteins associated with the anti-AML activity of 5-(1-hydroxyethyl)-dihydro-2-furanone. (**A**) GO enrichment analysis of the common target proteins involved in the predicted anti-AML activity of 5-(1-hydroxyethyl)-dihydro-2-furanone. (**B**) KEGG pathway enrichment analysis of the candidate target proteins related to AML. Enriched terms and pathways were considered statistically significant at *p* < 0.05.

To further elucidate the potential anti-AML mechanisms of 5-(1-hydroxyethyl)-dihydro-2-furanone, the 91 overlapping target proteins were subjected to Gene Ontology (GO) enrichment analysis using the DAVID online database. GO analysis provides functional annotation of genes or proteins in terms of biological process (BP), cellular component (CC), and molecular function (MF) [32]. A total of 357 GO terms were enriched, including 252 BP terms, 49 CC terms, and 56 MF terms. As shown in Figure 6A, the top 10 significantly enriched terms in each GO category were selected to represent the major biological functions associated with these target proteins. The enriched BP terms were mainly related to signal transduction, positive regulation of transcription by RNA polymerase II, apoptotic process, proteolysis, and protein phosphorylation. The CC terms primarily involved cytosol, cytoplasm, plasma membrane, nucleus, and extracellular exosome. The MF terms were mainly associated with protein binding, metal ion binding, identical protein binding, ATP binding, and enzyme binding activity. In addition, KEGG pathway enrichment analysis was performed to further explore the biological pathways associated with 91 target proteins. As shown in Figure 6B, a total of 120 KEGG pathways were enriched, of which the top 20 pathways were identified as the most relevant to the predicted anti-AML activity of 5-(1-hydroxyethyl)-dihydro-2-furanone. Among these, metabolic pathways and pathways in cancer were significantly enriched with the highest gene counts. Furthermore, apoptosis, MAPK signaling pathway, and PI3K/Akt signaling pathway were also prominently enriched, suggesting that the inhibitory effects of 5-(1-hydroxyethyl)-dihydro-2-furanone on leukemic cell proliferation may be mediated through the activation or suppression of these signaling pathways.

### 2.8. Anti-Inflammatory Activity of AE EtOAc No. 003 and the Ternary Mixture

**Figure 7:**
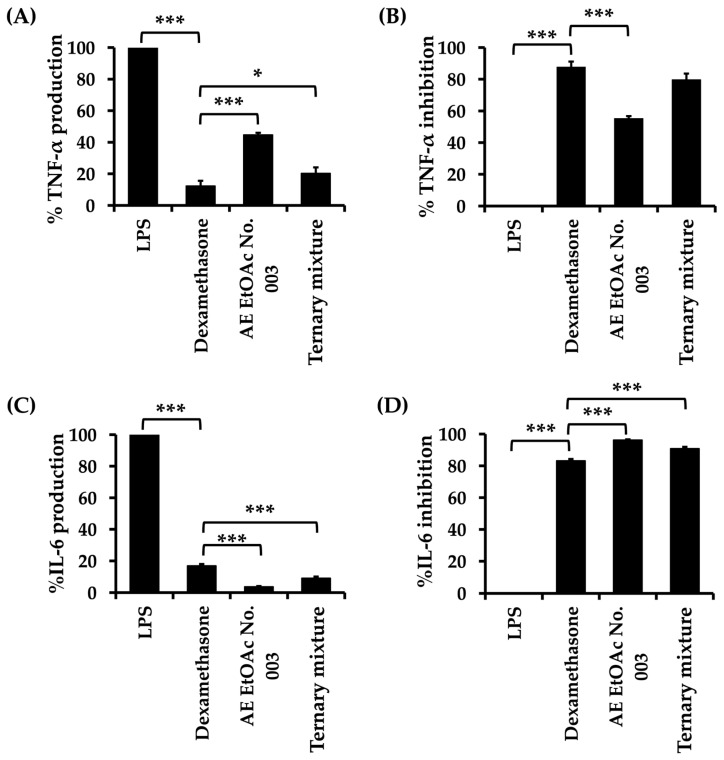
Anti-inflammatory activity of AE EtOAc No. 003 and the ternary mixture in LPS-stimulated RAW264.7 cells. The upper figures show (**A**) TNF-α production and (**B**) TNF-α inhibition, while the lower figures show (**C**) IL-6 production and (**D**) IL-6 inhibition. Data are expressed as mean ± SD of triplicate experiments (*n* = 3). Asterisks (*) indicate statistically significant differences between AE EtOAc No. 003 or the ternary mixture and dexamethasone (positive control) (* *p* < 0.05 and *** *p* < 0.001).

**Figure 8:**
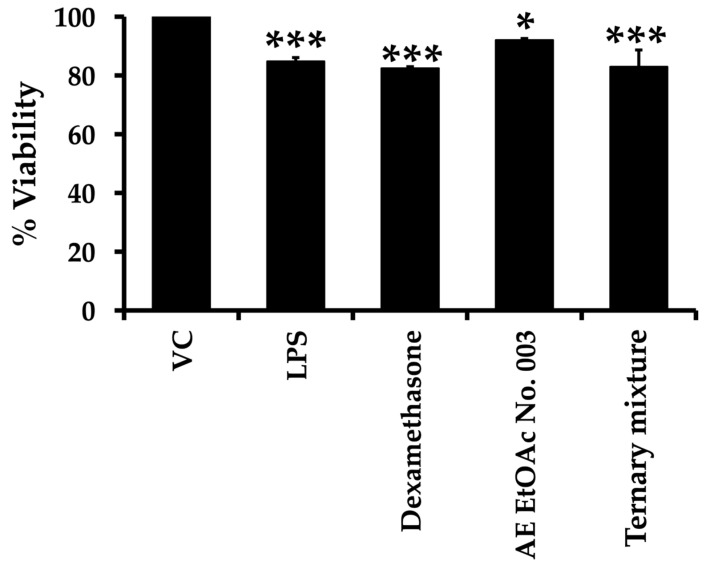
Effects of dexamethasone (positive control), AE EtOAc No. 003, and the ternary mixture on RAW264.7 cell viability and TNF-α and IL-6 production. Data are presented as the mean ± SD of triplicate experiments (*n* = 3). Asterisks (*) indicate statistically significant differences compared with the vehicle control (* *p* < 0.05 and *** *p* < 0.001).

RAW264.7 murine macrophage cell line was used as an in vitro model to investigate the anti-inflammatory effects of AE EtOAc No. 003 and the ternary mixture by assessing their ability to suppress the production of inflammatory mediators. Tumor necrosis factor-α (TNF-α) and interleukin-6 (IL-6) were selected as representative pro-inflammatory markers and quantified using enzyme-linked immunosorbent assay (ELISA) kits. Initially, the cytotoxic effects of AE EtOAc No. 003 and the ternary mixture on RAW264.7 cells were evaluated using the MTT assay. As summarized in Supplementary Table S5 and Figure S5, AE EtOAc No. 003 and the ternary mixture exhibited minimal cytotoxicity toward RAW264.7 macrophages at the concentration used, with cell viability remaining above 80%. These results support the use of these concentrations for subsequent anti-inflammatory assays without confounding effects from cytotoxicity. The IC_20_ values of AE EtOAc No. 003 and the ternary mixture were 26.82 ± 3.40 and 8.24 ± 0.76 µg/mL, respectively. Dexamethasone (1 µg/mL) was used as a positive control. As illustrated in Figure 7, dexamethasone, AE EtOAc No. 003 and the ternary mixture significantly reduced TNF-α and IL-6 production in lipopolysaccharide (LPS)-stimulated RAW264.7 cells. Notably, the anti-inflammatory activity of the ternary mixture was comparable to that of dexamethasone. In addition, cell viability following these treatments was further assessed using the MTT assay. As shown in Figure 8, dexamethasone, AE EtOAc No. 003 and the ternary mixture did not markedly affect RAW264.7 cell viability, with more than 80% viable cells compared with vehicle control (VC). Collectively, these results indicate that AE EtOAc No. 003 and the ternary mixture exert potent anti-inflammatory activity by suppressing pro-inflammatory cytokine production without causing significant cytotoxicity in RAW264.7 cells.

### 2.9. Effect of AE EtOAc No. 003 and the Ternary Mixture on Cell Cycle Distribution

**Figure 9:**
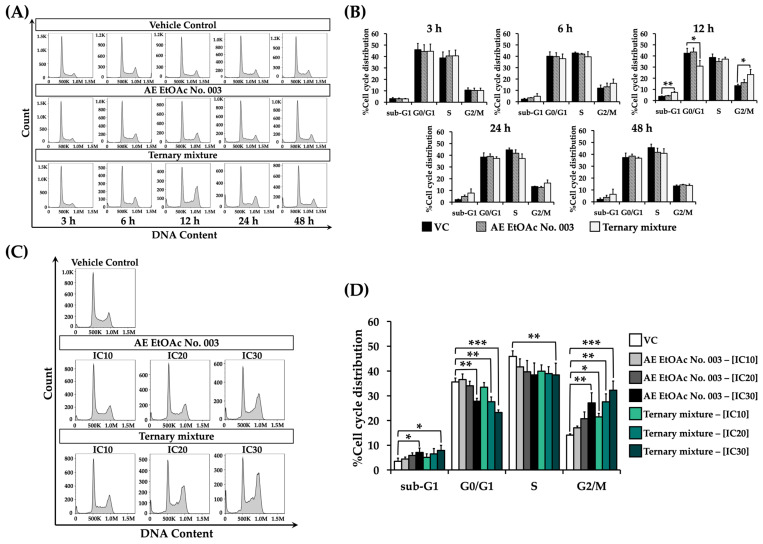
Cell cycle distribution of KG-1a cells following treatment with AE EtOAc No. 003 and the ternary mixture. KG-1a cells were treated with AE EtOAc No. 003 and the ternary mixture under different experimental conditions. (**A**) Representative histograms and (**B**) corresponding percentages of cell cycle distribution of KG-1a cells treated with AE EtOAc No. 003 or the ternary mixture at IC_20_ concentrations for various time points (3, 6, 12, 24, and 48 h). (**C**) Representative histograms and (**D**) corresponding percentages of cell cycle distribution in KG-1a cells treated with AE EtOAc No. 010 or the ternary mixture at different concentrations (IC_10_, IC_20_, and IC_30_) for 12 h. Panels (**A**,**C**) show representative flow cytometry histograms, while panels (**B**,**D**) present quantitative analysis from three independent experiments (*n* = 3). Data are expressed as the mean ± SD. * *p* < 0.05, ** *p* < 0.01, and *** *p* < 0.001 compared with vehicle control group (VC).

**Figure 10:**
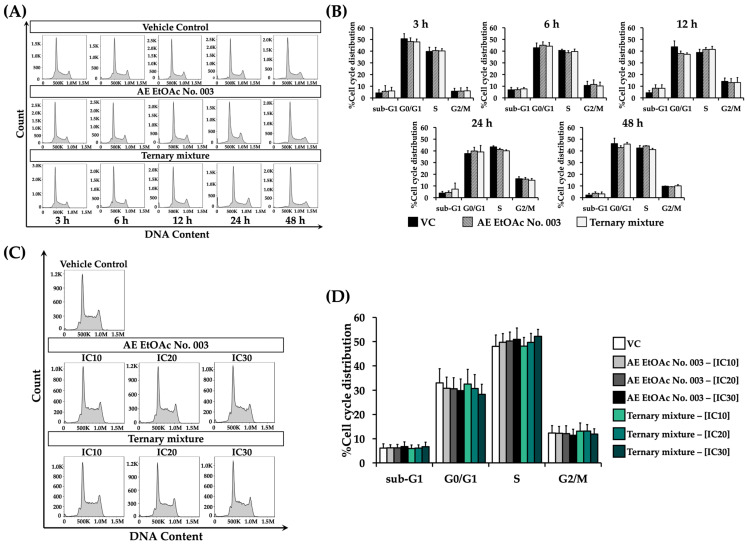
Cell cycle distribution of EoL-1 cells following treatment with AE EtOAc No. 010 and the ternary mixture. EoL-1 cells were treated with AE EtOAc No. 010 or the ternary mixture under different experimental conditions. (**A**) Representative histograms and (**B**) corresponding percentages of cell cycle distribution of EoL-1 cells treated with AE EtOAc No. 003 or the ternary mixture at IC_20_ concentrations for various time points (3, 6, 12, 24, and 48 h). (**C**) Representative histograms and (**D**) corresponding percentages of cell cycle distribution of EoL-1 cells treated with AE EtOAc No. 003 or the ternary mixture at different concentrations (IC_10_, IC_20_, and IC_30_) for 12 h. Panels (**A**,**C**) show representative flow cytometry histograms, while panels (**B**,**D**) present quantitative analysis from three independent experiments (*n* = 3). Data are expressed as the mean ± SD.

To evaluate the mechanisms underlying the inhibitory effects of AE EtOAc No. 003 and the ternary mixture on leukemic cell proliferation, the effects of these treatments on cell cycle distribution were first analyzed in KG-1a and EoL-1 cells using flow cytometry with propidium iodide (PI) staining. Both cell lines were treated with AE EtOAc No. 003 or the ternary mixture at their respective IC_20_ concentrations for 3, 6, 12, 24, and 48 h. As shown in Figure 9A,B, KG-1a cells exhibited a marked increase in the G2/M phase population after 12 h of treatment with AE EtOAc No. 003, which was more pronounced following treatment with the ternary mixture, compared with the vehicle control (VC). This increase subsequently declined with prolonged incubation times. In EoL-1 cells, a slight increase in sub-G1 and S phase populations was observed at 12 h compared with other time points (Figure 10A,B). Based on these findings, an incubation time of 12 h was selected for further analysis of concentration-dependent effects on cell cycle distribution. KG-1a and EoL-1 cells were then treated with AE EtOAc No. 003 or the ternary mixture at IC_10_, IC_20_, and IC_30_ concentrations for 12 h. In KG-1a cells, a concentration-dependent accumulation of cells in G2/M phase was observed following treatment with both AE EtOAc No. 003 and the ternary mixture (Figure 9C,D). In contrast, EoL-1 cells exhibited a modest but dose-dependent arrest in the S phase after treatment with AE EtOAc No. 003 and the ternary mixture (Figure 10C,D). Collectively, these results indicate that AE EtOAc No. 003 and the ternary mixture inhibit leukemic cell proliferation by inducing cell cycle arrest at G2/M of KG-1a cells and S phase of EoL-1 cells.

### 2.10. Effects of AE EtOAc No. 003 and the Ternary Mixture on Leukemic Cell Apoptosis

**Figure 11:**
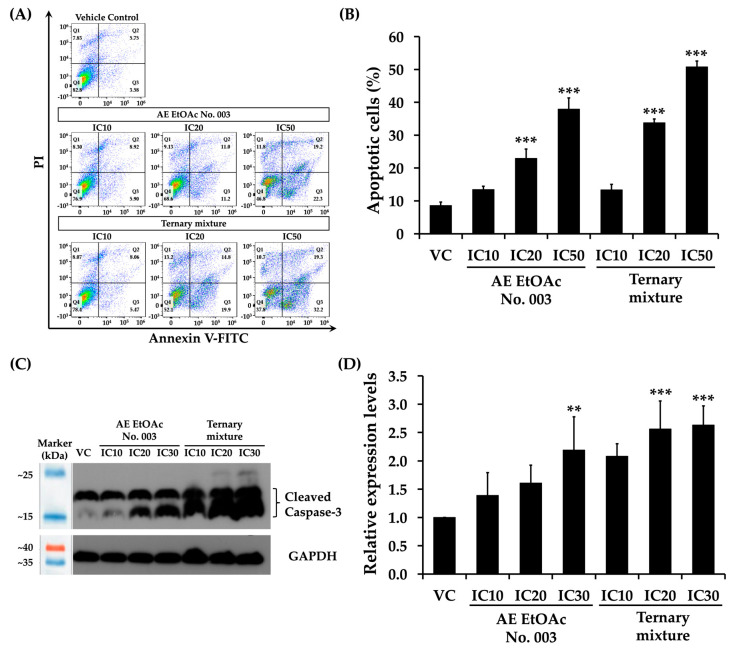
Effects of AE EtOAc No. 010 and the ternary mixture on apoptosis induction in KG-1a cells. (**A**,**B**) Apoptosis analysis of KG-1a cells treated with various concentrations of AE EtOAc No. 003 or the ternary mixture for 48 h, as determined by flow cytometry using Annexin V-FITC/PI staining. (**C**,**D**) Western Blot analysis of cleaved caspase-3 in KG-1a cells following treatment with AE EtOAc No. 003 or the ternary mixture for 48 h. Band intensities were quantified by scanning densitometry, and GAPDH was used as the internal control. Panels (**A**,**C**) show representative images, while panels (**B**,**D**) present quantitative data from three independent experiments (*n* = 3). Data are expressed as the mean ± SD. ** *p* < 0.01 and *** *p* < 0.001 compared with the vehicle control group (VC).

**Figure 12:**
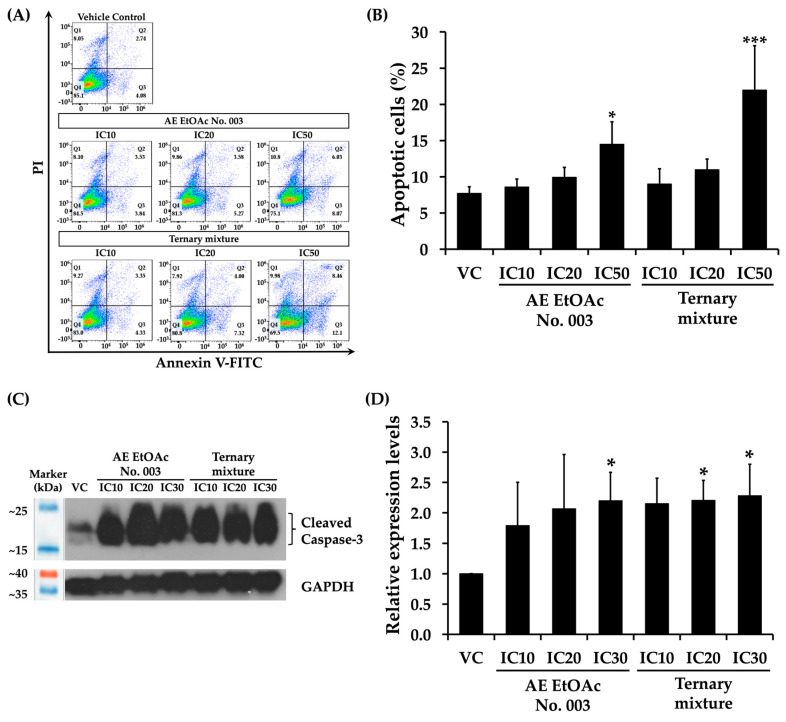
Effects of AE EtOAc No. 003 and the ternary mixture on apoptosis induction in EoL-1 cells. (**A**,**B**) Apoptosis analysis of EoL-1 cells treated with various concentrations of AE EtOAc No. 010 and the ternary mixture for 48 h, as determined by flow cytometry using Annexin V-FITC/PI staining. (**C**,**D**) Western Blot analysis of cleaved caspase-3 expression in EoL-1 cells following treatment with AE EtOAc No. 003 and the ternary mixture for 48 h. Band intensities were quantified by scanning densitometry, and GAPDH was used as the internal control. Panels (**A**,**C**) show representative images, while panels (**B**,**D**) present quantitative data from three independent experiments (*n* = 3). Data are expressed as the mean ± SD. * *p* < 0.05 and *** *p* < 0.001 compared with the vehicle control group (VC).

The identification of caspase-3 as a hub protein in PPI network and the enrichment of apoptosis-related pathways in the KEGG analysis, together with the observed increase in the sub-G1 population of KG-1a cells after 48 h of treatment with AE EtOAc No. 003 and the ternary mixture, prompted further investigation into the pro-apoptotic effects of this crude fractional extract and its active compound in leukemic cells. KG-1a and EoL-1 cells were treated with various concentrations (IC_10_, IC_20_, and IC_50_) of AE EtOAc No. 003 or the ternary mixture for 48 h, followed by Annexin V-FITC/PI staining and analysis by flow cytometry. As shown in Figure 11A,B, treatment with AE EtOAc No. 003 and the ternary mixture induce apoptotic cell death in KG-1a cells. Similarly, increased apoptosis was observed in EoL-1 cells following treatment (Figure 12A,B). The proportion of apoptotic cells increased in a concentration-dependent manner in both cell lines. In addition, the expression levels of cleaved caspase-3, a key effector protein in the intrinsic apoptotic pathway, were markedly increased in both KG-1a (Figure 11C,D) and EoL-1 cells (Figure 12C,D) in a dose-dependent manner following treatment with AE EtOAc No. 003 and the ternary mixture. These results were consistent with the flow cytometric analysis of apoptosis. Collectively, these findings indicate that AE EtOAc No. 003 and the ternary mixture suppress leukemic cell growth by inducing apoptosis.

## 3. Discussion

Acute myeloid leukemia (AML) is characterized by the accumulation of undifferentiated blast cells in the bone marrow, resulting in impaired normal hematopoiesis [3]. Epidemiological studies have reported a continuous increase in both the incidence and mortality rates of AML worldwide [1]. Although chemotherapy remains the mainstay of AML treatment, a substantial proportion of patients fail to achieve durable remission due to treatment-related toxicities and disease relapse [11]. Disease recurrence is frequently attributed to genetic alterations and the persistence of leukemic stem cells (LSCs), which possess self-renewal capacity and resistance to conventional chemotherapeutic agents [6]. Consequently, alternative therapeutic strategies, including medicinal plants, have attracted increasing interest as sources of bioactive compounds with antileukemic activity and minimal toxicity toward normal cells. In the present study, *A. evecta* demonstrated notable anti-leukemic potential. Among all crude fractional extracts, AE EtOAc No. 003 exhibited the greatest cytotoxic activity against KG-1a and EoL-1 cells while exerting only mild toxicity toward normal PBMCs. The observed variability in cytotoxic activity among *A. evecta* extracts from different geographical sources is likely influenced by environmental and edaphic factors that affect secondary metabolite biosynthesis, including soil composition, microbial communities, particle size, heavy metal content, pH, organic matter content, phytoremediation process, and agro-climatic conditions [33,34]. The biological relevance of the active fraction is supported by its ability to suppress leukemic cell proliferation, induce apoptosis, cause cell cycle arrest, and modulate WT1 expression. Structurally, this bioactive fraction was characterized as a ternary mixture composed of *epi*-osmundalactone (5,6-dihydro-5-hydroxy-6-methyl-2H-pyran-2-one), 5-(1-hydroxyethyl)-dihydro-2-furanone, and 5-(1-hydroxyethyl)-2(5H)-furanone, in a 2:2:1 ratio, as determined by NMR analysis. *Epi*-osmundalactone has previously been reported as a dual-lactone structure in association with angiopterolactone [35,36], and has also been isolated from *A. esculenta* Ching [24]. While angiopteroside has been widely studied for anti-bacterial [26] and anti-viral effects [28], the biological activity of (–)-*epi*-osmundalactone remain relatively underexplored. Several biological activities observed in this study are consistent with previous reports on Angiopteris-derived compounds. Notably, Lamichhane R. and colleagues demonstrated that (–)-*epi*-osmudalactone and angiopteroside from *A. helferiana* C.Presl exhibited anti-adipogenic activity and inhibited nitrite production, suggesting potential anti-inflammatory properties [27]. In addition, furanone derivatives have been extensively reported to possess diverse biological activities, including anti-inflammatory, anticancer, anti-microbial, and analgesic effects [37]. Synthetic derivatives of 2-furanone and 2(5H)-furanone, which share core structural similarity with the furanone components identified in the ternary mixture, have demonstrated cytotoxic activity against a broad spectrum of cancer cell lines, including leukemia and solid tumors [38,39,40]. Collectively, these findings support the notion that *epi*-osmundalactone and the associated furanone compounds may contribute synergistically to the observed anti-leukemic activity. However, in the present study, the observed biological effects cannot be attributed to a single constituent within the ternary mixture and may arise from the activity of one dominant component, additive effects, or synergistic interactions among the three co-eluting compounds.

Chronic inflammation plays a critical role in tumor initiation and progression. Elevated levels of TNF-α and IL-6 have been associated with tumor angiogenesis, invasion, and metastasis, and are closely linked to malignant progression [41,42]. In the present study, the ternary mixture. Compared with the positive control, dexamethasone, the ternary mixture significantly suppressed TNF-α and IL-6 production in LPS-stimulated RAW264.7 macrophages without inducing marked cytotoxicity, suggesting a genuine anti-inflammatory effect that may be relevant to its potential role in cancer prevention.

WT1 is a well-established biomarker of measurable residual disease in AML and is frequently overexpressed in primary leukemic blasts compared with the normal hematopoietic stem and progenitor cells. Elevated WT1 expression has been associated with leukemic cell proliferation, disease progression, poor prognosis, and relapse in AML patients [43]. Conversely, suppression of WT1 expression has been reported to inhibit leukemic cell growth and is associated with improved clinical outcomes [31]. Experimental knockdown or silencing of *WT1* expression induces apoptosis, alters cell cycle progression, and suppress leukemic proliferation [29,44]. Accordingly, several anticancer agents, including curcumin [45,46], resveratrol [47], shikonin [48], the HSP90 inhibitor 17-AAG [49], and WP1130 [50], have been shown to exert anti-leukemic effects through modulation of WT1-related pathways. In the present study, however, WT1 modulation was not uniformly observed across the two AML cell lines examined. AE EtOAc No. 003 and the ternary mixture did not significantly alter WT1 expression in KG-1a cells, despite a marked reduction in cell number following treatment, indicating that WT1-independent mechanisms, such as apoptosis induction and cell cycle arrest, may predominate in this cellular context. In contrast, the ternary mixture significantly downregulated WT1 expression in EoL-1 cells in a dose-dependent manner, which correlated with reduced cell proliferation. Taken together, these findings suggest that WT1 is not a universal primary target of the ternary mixture across AML models but rather a context-dependent mediator whose contribution varies among leukemic subtypes. While WT1-associated mechanisms appear to contribute to growth inhibition in EoL-1 cells, alternative WT1-independent pathways likely underline the antileukemic effects observed in KG-1a cells.

Molecular docking analysis provided structural support for the anti-leukemic activity of the isolated furanone compounds. Among them, 5-(1-hydroxyethyl)-dihydro-2-furanone exhibited the highest binding affinity to the WT1-DNA complex (−6.3 kcal/mol), forming stable hydrogen-bond interactions with Arg87 and adjacent DNA nucleotides (DG129/DC151). Given the critical role of WT1 in driving AML progression and poor clinical prognosis, binding at the WT1 protein-DNA interface may interfere with its transcriptional regulatory function [51]. This observation is consistent with our experimental findings showing a significant downregulation of WT1 expression in EoL-1 cells following treatment with the ternary mixture. In addition, favorable binding scores toward ERK1 and ERK2 further support the network pharmacology prediction that MAPK signaling may be involved in the anti-proliferative effects of this compound. WT1 has been reported to positively regulate MAPK/ERK signaling, thereby promoting leukemic cell survival [52]. Accordingly, the simultaneous targeting of WT1 and MAPK-related proteins may contribute to the observed inhibitory effects on AML cell proliferation. Collectively, these findings suggest that 5-(1-hydroxyethyl)-dihydro-2-furanone exhibited the highest binding affinity toward the WT1-DNA complex and MAPK-related proteins, supporting its potential involvement in the coordinated modulation of WT1-dependent transcriptional regulation and MAPK/ERK signaling pathways. This component may therefore play a prominent role in the observed anti-leukemic activity; however, experimental validation is required to confirm this hypothesis.

Based on the molecular docking analysis, the interactions and binding energies between WT1 protein and the compounds constituting the ternary mixture were evaluated. In addition, network pharmacology analysis was employed to predict and elucidate the underlying molecular mechanisms of 5-(1-hydroxyethyl)-dihydro-2-furanone in the treatment of AML. The analysis identified 91 overlapping target proteins associated with both AML and 5-(1-hydroxyethyl)-dihydro-2-furanone. Among these targets, AKT1, caspase-3, EGFR, ERBB2, and caspase-8 were identified as key proteins potentially mediating the anti-proliferative activity of 5-(1-hydroxyethyl)-dihydro-2-furanone. Furthermore, GO and KEGG pathway enrichment analyses revealed that the MAPK signaling pathway and apoptosis-related pathways were significantly involved in the predicted anti-AML activity of this compound. These findings support our experimental results demonstrating that 5-(1-hydroxyethyl)-dihydro-2-furanone inhibits leukemic cell proliferation primarily through the induction of apoptotic cell death. The MAPK signaling pathway plays a critical role in regulating cell proliferation, survival and differentiation in both normal and malignant cells, including leukemic cells. Aberrant activation of the MAPK pathway in AML has been reported to contribute to sustained proliferation, resistance to apoptosis, and impaired differentiation of leukemic blast cells [53]. In previous study by Li XY. et al. (2014), expression of WT1 isoforms in U937 leukemic cells markedly upregulated signaling molecules involved in the JAK-STAT and MAPK pathways, resulting in enhanced cell migration, suppression of apoptosis, and increased colony formation [54]. Integrating these findings with our results on WT1 expression and network pharmacology prediction suggests that, in addition to directly inducing apoptosis, downregulation of WT1 expression may contribute to the anti-proliferative effects of 5-(1-hydroxyethyl)-dihydro-2-furanone found that in addition to inducing cell apoptosis, repressing of WT1 expression might influence the inhibitory effect of leukemic cell proliferation of 5-(1-hydroxyethyl)-dihydro-2-furanone through modulating of MAPK signaling. Nevertheless, further in vitro and in vivo studies are required to comprehensively validate the involvement of these signaling pathways and to fully assess the therapeutic potential of 5-(1-hydroxyethyl)-dihydro-2-furanone in AML treatment.

Taken together, these in silico analyses (molecular docking and network pharmacology) provide hypothesis-generating insights into potential molecular targets and signaling pathways associated with the observed biological effects; however, experimental validation is required to confirm the involvement of these predicted mechanisms.

Consistent with these mechanistic insights, the ternary mixture suppressed the proliferation of KG-1a cells predominantly through the induction of apoptosis and cell cycle arrest at the G2/M phase, whereas its effects on EoL-1 cells appeared to involve multiple mechanisms. In EoL-1 cells, the ternary mixture induced apoptotic cell death, caused a modest arrest in the S phase of cell cycle, and significantly downregulated WT1 expression in a dose-dependent manner. These observations are consistent with the corresponding changes in cell proliferation rates. The differential responses observed between KG-1a and EoL-1 cells suggest that the compounds constituting the ternary mixture may exert cell line-specific effects, potentially reflecting phenotypic and molecular heterogeneity among leukemic cell types [55,56]. Although further purification and individual evaluation of *epi*–osmundalactone and the two furanone derivatives present in the ternary mixture are required to delineate their distinct biological activities, the present study supports the potential of *A. evecta*-derived furanone compounds as promising, biologically driven lead candidates for anti-leukemic drug development.

## 4. Materials and Methods

### 4.1. Plant Materials and Extraction

*A. evecta* rhizomes were collected from 12 locations in Thailand, including nine herbal shops and three natural habitats, during April 2020. Plants materials were taxonomically identified and authenticated by Angkhana Inta (Chiang Mai University, Thailand), Sukanda Chaiyong, Trinnakorn Katekulaphan, and Methee Rungrojsakul (Chandrakasem Rajabhat University, Thailand). Voucher specimens (WP6607, WP6608, and WP6609) were deposited at the Herbarium of the Queen Sirikit Botanical Garden. Briefly, all dried rhizomes of *A. evecta* were ground into a fine powder and subsequently extracted with solvents of increasing polarity, namely as *n*–hexane (*n*–Hex), ethyl acetate (EtOAc) and ethanol (EtOH), respectively. *A. evecta* was extracted with each solvent by maceration for 72 h, with drying intervals of 24 h between each solvent. Subsequently, each collected extract was followed by filtration with Whatman paper, solvent evaporation, and concentration under reduced pressure. 25 mg/mL stock solution of crude fractional extracts was prepared by dissolving in 100% dimethyl sulfoxide (DMSO). These crude fractional extracts were subsequently subjected to biological evaluation for their inhibitory effects on leukemic cell proliferation.

### 4.2. Chemical Materials

3-(4,5-Dimethylthiazol-2-yl)-2,5-diphenyltetrazolium bromide (MTT) was purchased from Sigma-Aldrich (St. Louis, MO, USA). Dulbecco’s Modified Eagle’s Medium (DMEM), Iscove’s Modified Dulbecco’s Medium (IMDM), RPMI–1640 medium, Dulbecco’s Phosphate-Buffered Saline (DPBS), Penicillin (10,000 U/mL)/Steptomycin (10,000 μg/mL) and 200 mM L-Glutamine were purchased from GIBCO Invitrogen™ (Grand Island, NY, USA). Fetal bovine serum (FBS) was obtained from Capricorn Scientific (Ebsdorfergrund, Germany). Spectra™ Multicolor Broad Range Protein Ladder was purchased from Thermo Fisher Scientific (Waltham, MA, USA). 30% T Acrylamide/Bis-acrylamide (37.5:1) solution was purchased from PanReact AppliChem (Darmstadt, Germany). 1 M Tris-HCl, pH 8 solution, and 0.5 M Tris-HCl, pH 6.8 solution were purchased from Bio-Rad Laboratories (Richmond, CA, USA). Ethanol (EtOH), ethyl acetate (EtOAc), *n*–hexane (*n*–Hex), and dimethyl sulfoxide (DMSO) were purchased from Labscan (Dublin, Ireland). Silica gel 60 was purchased from Merck (Darmstadt, Germany).

### 4.3. Column Chromatography and Characterization

The bioactive crude fractional extract of *A. evecta* was purified using a combination of normal-phase column chromatography and reverse phase high polarity liquid chromatography (RP-HPLC). For the initial fractionation, silica gel grade 60 was used as the stationary phase and packed in a column. Crude fractional extract (5.0 g) was added to the top of the column and eluted with mixtures of *n*-hexane and EtOAc employing a stepwise gradient with increasing polarity (*n*-Hex:EtOAc = 100:0 to 0:100). Fractions were collected in test tubes (8 mL/tubes) and monitored with thin layer chromatography (TLC). Fractions with similar TLC patterns were pooled together, observed using TLC, and characterized in terms of their chemical structures using 500 MHz nuclear magnetic resonance (NMR) spectroscopy (Bruker, Fällanden, Switzerland) at the Faculty of Science, Chiang Mai University, or with a JEOL (Tokyo, Japan) JNM-ECA 500 MHz spectrometer at the Department of Materials and Life Sciences, Sophia University. Using electrospray ionization–mass spectrometry (ESI-MS) spectra with time-of-flight (TOF) detection for high-resolution measurements, spectra were recorded on a JEOL JMS-T100LC instrument at Sophia University.

Further purification and isolation of the dominate purified compound, subsequently identified as a ternary mixture that co-eluted as a single chromatographic peak, were achieved by reverse-phase high performance liquid chromatography (RP-HPLC) at Sophia University. The chromatographic separation was performed using a Shimadzu RP-HPLC system (Kyoto, Japan) equipped with a quaternary pump (LC-20A, ×2 units), a degasser, a thermostatic autosampler (SIL-20AC), a photodiode array detector (SPD-20AV), and a column oven (CTO-20AC). Separation was carried out on a C18 reversed-phase column (5C18-AR-II, 250 mm) fitted with a C18 guard column (5C18-MS-II, 20 mm). The column temperature was maintained at 40 °C, and the injection volume was 0.02 mL. The mobile phase consisted of water (H_2_O; B) and acetonitrile (ACN; A), delivered at a constant flow rate of 1.0 mL/min over a total run time of 25 min. The gradient program was as follows: 100% B decreasing to 20% B over the first 15 min, followed by re-equilibration to 100% B for the remaining 10 min. Elution was monitored at 210 and 230 nm, and fractions corresponding to the co-eluting ternary mixture were collected.

*A. evecta* crude fractional extracts and purified compounds were stored at −20 °C. Crude fractional extracts and purified compounds were dissolved in DMSO to obtain the working concentration (25 mg/mL) and stored at −20 °C for later use.

### 4.4. Network Pharmacology

#### 4.4.1. Identification of Potential Targets of the Active Compound from *A. evecta* in AML

The chemical structure of the active compound from *A. evecta* was obtained from the PubChem database (https://pubchem.ncbi.nlm.nih.gov/, accessed on 8 October 2025). Potential protein targets of the active compound were predicted using the PharmMapper and SwissTargetPrediction databases. AML-related disease targets were retrieved from the GeneCards and DisGeNET database. After removing duplicate protein targets from dataset, the overlapping targets between the active compound and AML were identified using the Venny 2.1 online tool (https://bioinfogp.cnb.csic.es/tools/venny/, accessed on 8 October 2025) and were considered as the predicted therapeutic targets of the active compound against AML.

#### 4.4.2. Network Construction

Protein–protein interaction (PPI) networks of the overlapping target genes were constructed using the STRING database (https://string-db.org/, accessed on 10 October 2025). The resulting PPI network was visualized and analyzed using Cytoscape software (version 3.10.2). Key target genes were screened based on degree centrality values. In addition, Gene Ontology (GO) functional annotation and Kyoto Encyclopedia of Genes and Genomes (KEGG) pathway enrichment analyses of the overlapping target genes were performed using the DAVID online platform (https://davidbioinformatics.nih.gov/, accessed on 10 October 2025). Enriched GO terms and KEGG pathways with a *p*-value < 0.05 were considered statistically significant.

### 4.5. Cells and Cell Culture Conditions

The leukemic cell lines used in this study included KG-1a, a leukemic stem cell–like cell line enriched in stem cell populations, and EoL-1, a human promyelocytic leukemia cell line (ATCC, Manassas, VA, USA). RAW264.7, a murine macrophage cell line, was used as an in vitro model to evaluate the anti-inflammatory activity of *A. evecta* crude fractional extracts and the active compound. Peripheral blood mononuclear cells (PBMCs) were isolated from healthy donors by Ficoll–Hypaque density-gradient centrifugation using Histopaque^®^-1077 (Sigma-Aldrich, Saint Louis, MO, USA). The KG-1a cell line was cultured in Iscove’s Modified Dulbecco’s Medium (IMDM) supplemented with 20% fetal bovine serum (FBS), 100 units/mL penicillin, and 100 µg/mL streptomycin. The EoL-1 cell line and PBMCs were maintained in RPMI-1640 medium supplemented with 10% FBS, 1 mM L-glutamine, 100 units/mL penicillin, and 100 µg/mL streptomycin. RAW264.7 cells were cultured in Dulbecco’s Modified Eagle’s Medium (DMEM) supplemented with 10% FBS, 1 mM L-glutamine, 100 units/mL penicillin, and 100 µg/mL streptomycin. All cells were maintained at 37 °C in a humidified atmosphere containing 5% CO_2_. The use of human PBMCs in this study was approved by the Human Research Ethics Unit of the Faculty of Associated Medical Sciences, Chiang Mai University (study code No. 196/2022; approval date: 27 June 2022).

### 4.6. Cytotoxicity Analysis Using MTT Assay

Cells were seeded in 96-well plates and cultured for 24 h before the treatment with various concentrations of *A. evecta* crude fractional extracts or purified compounds (0–100 µg/mL) for 48 h. Following incubation, cytotoxicity was assessed using the MTT (3-(4,5-dimethylthiazol-2-yl)-2,5-diphenyltetrazolium bromide) assay, as previously described [47]. Briefly, 15 µL of MTT solution (5 mg/mL) was added to each well and incubated for 4 h at 37 °C. Subsequently, dimethyl sulfoxide (DMSO) was added to dissolve the formazan crystals, and the plates were gently agitated to ensure complete solubilization. Absorbance was measured at 578 nm using a microplate reader (Beckman Coulter DTX880, Fullerton, CA, USA), with a reference wavelength of 630 nm. Cell viability was calculated according to Equation (1), with the absorbance of DMSO-treated vehicle control cells defined as 100% viability:

(1) $$\%\;\mathrm{Cell}\;\mathrm{viability}\;=\;\frac{\mathrm{Mean}\;\mathrm{absorbance}\;\mathrm{in}\;\mathrm{test}\;\mathrm{well}}{\mathrm{Mean}\;\mathrm{absorbance}\;\mathrm{in}\;\mathrm{vehicle}\;\mathrm{control}\;\mathrm{well}}\;\times \;100$$

Data from three independent experiments were used to generate dose–response curves. Half-maximal inhibitory concentration (IC_50_) values were calculated and are presented as the mean ± standard deviation (SD).

### 4.7. Selectivity Index

The selectivity index (SI) is commonly reported as a quantitative parameter to evaluate the preferential cytotoxicity of a compound toward cancer cells relative to normal cells. It is defined as the ratio of the half-maximal inhibitory concentration (IC_50_) obtained in normal cells to that obtained in cancer cells, and is widely used as an indicator of anticancer selectivity [57,58]. An SI value greater than 3 is generally considered to indicate desirable selectivity toward cancer cells. In this study, the selectivity index (SI) of the active compounds and reference chemotherapeutic drugs was calculated using Equation (2):

(2) $$\mathrm{Selectivity}\;\mathrm{index}\;=\;\frac{{\mathrm{IC}}_{50\;N}}{{\mathrm{IC}}_{50\;C}}$$

where IC_50 N_ represents the IC_50_ value determined in normal cells (PBMCs) and IC_50 C_ represents the IC_50_ determined in cancer cells (KG-1a and EoL-1 leukemic cell lines) under identical experimental conditions.

### 4.8. Trypan Blue Exclusion Assay

Following treatment, cell proliferation was evaluated using the trypan blue exclusion assay. Cell suspensions were mixed with 0.2% trypan blue solution, and viable (unstained) and nonviable (stained) cells were counted under a light microscope using a hemocytometer. The percentage of viable cells and the cell proliferation rate were subsequently calculated. All experiments were performed in triplicate.

### 4.9. Determination of Anti-Inflammatory Activity

The anti-inflammatory activity of *A. evecta* crude fractional extract and the ternary mixture was evaluated by measuring the production of pro-inflammatory cytokines, including IL-6 and TNF–α. The murine macrophage cell line RAW264.7 was used as an in vitro inflammation model. Cells were seeded at a density of 1 × 10^5^ cells/well in 24-well plates and cultured for 24 h. After incubation, the cells were pretreated with *A. evecta* crude fractional extract or the ternary mixture at their respective IC_20_ concentrations, or with dexamethasone (1 µg/mL) as a positive control, for 2 h. Inflammation was subsequently induced by stimulation with lipopolysaccharide (LPS) at a final concentration of 1 µg/mL for 24 h [59]. LPS-treated RAW264.7 cells were used as vehicle control, and cytokine production in this group was defined as 100%. Untreated cells and dexamethasone-treated cells served as negative and positive controls, respectively. Following treatment, culture supernatants were collected and analyzed for cytokine secretion using mouse IL-6 uncoated ELISA kits (Cat. no. 88-7064; Thermo Fisher Scientific, MA, USA) and mouse TNF-α uncoated ELISA kits (Cat. no. 88-7324; Thermo Fisher Scientific), according to the manufacturers’ instructions. Absorbance was measured at 450 nm with a reference wavelength of 570 nm using a microplate reader (Beckman Coulter DTX880, Fullerton, CA, USA). The inhibition of IL-6 and TNF-α secretion was calculated using Equation (3):

(3) $$\%\;\mathrm{Cytokine}\;\mathrm{inhibition}\;=\;\frac{A\;-\;B\;}{A}\;\times \;100$$

Here, A represents the mean absorbance of LPS-treated RAW264.7 cells without sample treatment, whereas B represents the mean absorbance of LPS-treated RAW264.7 cells in the presence of sample treatment. In addition, RAW264.7 cell viability was assessed using the MTT assay. All experiments were performed in triplicate.

### 4.10. Cell Cycle Distribution

KG-1a and EoL-1 cells were treated with *A. evecta* crude fractional extract or the ternary mixture under the indicated experimental conditions. After incubation, cells were harvested, washed twice with cold phosphate-buffered saline (PBS, pH 7.4), and fixed in 70% ice-cold ethanol for 2 h. Following fixation, the cells were washed with cold PBS (pH 7.4) and incubated with propidium iodide (PI) and RNase staining solution (1×; Abcam, Cambridge, UK) according to the manufacturer’s instructions. Cell cycle distribution was analyzed using a DxFLEX flow cytometer (Beckman Coulter, Fullerton, CA, USA). All experiments were performed in triplicate.

### 4.11. Apoptosis Analysis

Following treatments of *A. evecta* crude fractional extract or the ternary mixture with KG-1a and EoL-1 cells, cells were harvested and apoptosis was analyzed using the FITC Annexin V Apoptosis Detection Kit with propidium iodide (PI) (BioLegend, San Diego, CA, USA), according to the manufacturer’s instructions. Briefly, cell pellets were resuspended in 100 µL of Annexin V Binding Buffer. Subsequently, 5 µL of Annexin V-FITC and 10 µL of PI were added to each sample, gently mixed, and incubated for 15 min at room temperature in the dark. After incubation, 400 µL of Annexin V Binding Buffer was added to each sample. Apoptotic cells were analyzed using a DxFLEX flow cytometer (Beckman Coulter, Fullerton, CA, USA). All experiments were performed in triplicate.

### 4.12. Western Blot Analysis

KG-1a and EoL-1 cells were treated with *A. evecta* crude fractional extract or the ternary mixture under the indicated experimental conditions. Cells were harvested, and total protein was extracted using radioimmunoprecipitation assay (RIPA) buffer supplemented with a protease inhibitor cocktail. Protein concentrations were determined using the Pierce™ BCA Protein Assay Kit (Thermo Fisher Scientific, Waltham, MA, USA). Equal amounts of protein (50 µg) were separated by 12% sodium dodecyl sulfate–polyacrylamide gel electrophoresis (SDS–PAGE) and subsequently transferred onto polyvinylidene difluoride (PVDF) membranes. To prevent nonspecific binding, membranes were blocked with 5% (*w*/*v*) skim milk in Tris-buffered saline containing 0.1% Tween-20 (TBST) for at least 30 min at room temperature. The membranes were then incubated with the following primary antibodies: rabbit monoclonal anti-WT1 IgG (1:1000; Cell Signaling Technology, Danvers, MA, USA), rabbit monoclonal anti–cleaved caspase-3 (Asp175) (5A1E) IgG (1:1000; Cell Signaling Technology), and rabbit polyclonal anti-human GAPDH IgG (1:16,000; Santa Cruz Biotechnology, CA, USA) as a loading control. Incubations with anti-WT1 and anti–cleaved caspase-3 antibodies were performed overnight at 4 °C, whereas incubation with the anti-GAPDH antibody was carried out for 1 h at room temperature. After primary antibody incubation, membranes were washed with TBST to remove unbound antibodies. Membranes were subsequently incubated with a horseradish peroxidase (HRP)-conjugated anti-rabbit IgG secondary antibody (1:20,000; Promega, Madison, WI, USA). Immunoreactive protein bands were visualized using Luminata™ Forte Western HRP substrate (Merck, Darmstadt, Germany), and chemiluminescent signals were captured on X-ray films (Sakura, Tokyo, Japan). Band intensities were quantified using Quantity One 1-D Analysis Software version 4.6.6 (Bio-Rad, Hercules, CA, USA) and normalized to GAPDH expression levels. All experiments were performed in triplicate.

### 4.13. Docking Analysis

Molecular docking was utilized to evaluate the intermolecular interactions between the three compounds and the core target protein WT1. The 3D structures of the small-molecule ligands were obtained from the PubChem database (https://pubchem.ncbi.nlm.nih.gov, accessed on 19 September 2025), while the crystal structure of WT1 (PDB ID: 4R2E) was downloaded from the RCSB PDB (https://www.rcsb.org/, accessed on 19 September 2025). PyMOL software version 3.1 was used to remove water molecules and co-crystallized ligands from the protein structure, followed by hydrogen addition, charge calculation, and nonpolar hydrogen processing for both receptor and ligand using AutoDock Tools version v4.2.6. After defining the docking grid dimensions and algorithm parameters, molecular docking was performed using AutoDock Vina version v1.2.0. The docking conformations and interaction sites were visualized using Discovery Studio version 24.1 and PyMOL.

### 4.14. Statistical Analysis

All experiments were performed in triplicate independent experiments to ensure reproducibility. Data are presented as the mean ± standard deviation (SD). Statistical analyses were conducted using IBM SPSS Statistics software (version 22.0). Differences among groups were analyzed using one-way analysis of variance (ANOVA), followed by least significant difference (LSD) post hoc tests where appropriate. Statistical significance was defined as * *p* < 0.05, ** *p* < 0.01, and *** *p* < 0.001.

## 5. Conclusions

Among the 36 crude fractional extracts obtained from *A. evecta*, AE EtOAc No. 003 exhibited the strongest cytotoxic activity against KG-1a and EoL-1 leukemic cells. From this extract, the ternary mixture—comprising *epi*-osmundalactone, 5-(1-hydroxyethyl)-dihydro-2-furanone, and 5-(1-hydroxyethyl)-2(5H)-furanone in a ratio of 2:2:1—was identified as the major bioactive fraction with synergistic biological properties. The ternary mixture demonstrated potent anti-inflammatory activity by significantly suppressing TNF-α and IL-6 production in LPS-stimulated macrophages. Furthermore, both AE EtOAc No. 003 and the ternary mixture effectively inhibited leukemic cell proliferation through the induction of apoptosis and cell cycle arrest. Mechanistic investigations suggested that these effects may be mediated, at least in part, through modulation of apoptosis-related pathways and MAPK/AKT signaling. Collectively, the findings of this study indicate that *A. evecta* and its furanone-rich constituents represent promising natural sources for the development of novel lead compounds for the prevention of leukemogenesis and the treatment of leukemia. Further in-depth mechanistic studies and in vivo validation are warranted to fully elucidate their therapeutic potential.

## Figures and Tables

**Table 1:** % yields of crude fractional extracts from 12 sources of *A. evecta*.

*A. evecta* (Location)	Yield (% *w*/*w*)		
	*n*-Hex	EtOAc	EtOH
001 (Bangkok 1)	0.24	0.4	2.37
002 (Bangkok 2)	0.3	0.29	1.85
003 (Bangkok 3)	0.23	0.46	2.35
004 (Bangkok 4)	0.25	0.47	4.30
005 (Nonthaburi)	0.23	0.33	3.78
006 (Phitsanulok)	0.23	0.52	3.18
007 (Lopburi)	0.22	0.43	4.26
008 (Phuket)	0.23	0.29	1.83
009 (Chiang Mai 1)	0.26	0.29	1.58
010 (Chiang Mai 2)	0.23	0.48	4.97
011 (Nan)	0.17	0.48	4.90
012 (Tak)	0.26	0.57	3.80

**Table 2:** IC_50_ values (µg/mL) of crude fractional extracts from 12 sources of *A. evecta* in KG-1a and EoL-1 leukemic cells, and PBMCs.

Crude Fractional Extract (Location)	IC_50_ Values (Mean ± SD, µg/mL)		
	KG-1a	EoL-1	PBMCs
001 (Bangkok 1)			
Hex	>100	98.97 ± 12.78	ND
EtOAc	69.30 ± 11.96	42.41 ± 1.55	ND
EtOH	>100	>100	ND
002 (Bangkok 2)			
Hex	>100	>100	ND
EtOAc	>100	69.94 ± 3.44	ND
EtOH	>100	>100	ND
003 (Bangkok 3)			
Hex	>100	>100	ND
EtOAc	23.29 ± 3.78 *	13.29 ± 1.90 *	40.91 ± 6.17
EtOH	>100	>100	ND
004 (Bangkok 4)			
Hex	>100	>100	ND
EtOAc	39.66 ± 4.18	31.42 ± 1.04	ND
EtOH	>100	>100	ND
005 (Nonthaburi)			
Hex	>100	>100	ND
EtOAc	46.66 ± 3.16	36.35 ± 1.21	ND
EtOH	>100	>100	ND
006 (Phitsanulok)			
Hex	>100	77.31 ± 4.94	ND
EtOAc	42.79 ± 2.10	29.18 ± 1.18	ND
EtOH	>100	>100	ND
007 (Lopburi)			
Hex	>100	84.88 ± 7.15	ND
EtOAc	74.52 ± 5.10	37.37 ± 0.68	ND
EtOH	>100	>100	ND
008 (Phuket)			
Hex	>100	71.77 ± 6.17	ND
EtOAc	>100	75.11 ± 0.94	ND
EtOH	>100	>100	ND
009 (Chiang Mai1)			
Hex	>100	70.68 ± 5.25	ND
EtOAc	84.99 ± 5.15	52.07 ± 4.03	ND
EtOH	>100	>100	ND
010 (Chiang Mai 2)			
Hex	>100	78.11 ± 5.69	ND
EtOAc	49.81 ± 4.65	32.06 ± 1.70	ND
EtOH	>100	>100	ND
011 (Nan)			
Hex	>100	>100	ND
EtOAc	89.50 ± 2.89	41.85 ± 2.03	ND
EtOH	>100	>100	ND
012 (Tak)			
Hex	>100	86.74 ± 4.11	ND
EtOAc	>100	>100	ND
EtOH	>100	>100	ND

ND = not determined, * good cytotoxicity in each cell line. Results were expressed as mean ± SD of three independent experiments.

**Table 3:** IC_50_ values (µg/mL) and Selectivity index (SI) of the ternary mixture, isolated from AE EtOAc No. 003 (Purified compound 4), and doxorubicin in KG-1a and EoL-1 leukemic cells, and PBMCs.

Compound and Drug	IC_50_ Values (Mean ± SD)			Selectivity Index (SI)	
	KG-1a	EoL-1	PBMCs	KG-1a	EoL-1
Ternary mixture (Purified compound 4) (µg/mL)	9.73 ± 0.24	8.10 ± 0.73	51.36 ± 8.37	5.28	6.34
Doxorubicin (ng/mL)	1509.43 ± 89.78	1.30 ± 0.04	>1000	>0.66	>771.04

Results were expressed as mean ± SD of three independent experiments.

**Table 4:** Component-target molecular docking.

Compounds	Binding Energy (kcal/mol)							
	WT1	ERK1	ERK2	JNK1	JNK2	PIK3C3	PKCα	PKCγ
*epi*-Osmundalactone	−5.3	−6.0	−6.0	−5.1	−4.8	−5.4	−4.6	−4.1
5-(1-hydroxyethyl)-dihydro-2-furanone	−6.3	−5.6	−5.6	−4.6	−5.1	−5.4	−4.3	−3.8
5-(1-hydroxyethyl)-2(5H)-furanone	−6.2	−6.0	−6.0	−4.7	−4.6	−5.2	−4.3	−3.8

## Data Availability

The original contributions presented in the study are included in the article/Supplementary Materials, further inquiries can be directed to the corresponding authors.

## Supplementary Materials

The following supporting information can be downloaded at: https://www.mdpi.com/article/10.3390/ijms27031399/s1.
