# Supplementary material for: Isolation and Characterization of 5-(1-Hydroxyethyl)-Dihydro-2-Furanone from Angiopteris evecta with Potent Anti-Inflammatory and Anti-Leukemic Activities
Source: Int J Mol Sci. 2026 Jan 30;27(3):1399. doi: 10.3390/ijms27031399 (PMC12897760; doi:10.3390/ijms27031399)
Supplement: Supplementary file 1 [file ijms-27-01399-s001.zip › ijms-4093677-supplementary.pdf]

Supplementary data

Isolation and Characterization of 5-(1-hydroxyethyl)-dihydro-2-furanone from *Angiopteris evecta* with Potent Anti-Inflammatory and Anti-Leukemic Activities

Lapamas Rueankham [1], Natsima Viriyaadhammaa [1,2], Wenxian Yin [1], Yuanzhi Liu [3], Sawitree Chiampanichayakul [1,2,4], Methee Rungrojsakul [5], Trinnakorn Katekunlaphan [6], Siriporn Okonogi [4,7], Aroonchai Saiai [8], Arihiro Iwasaki [9], Christian Nanga Chick [10], Toyonobu Usuki [10,*] and Songyot Anuchapreeda [1,2,4,*]

[1] Department of Medical Technology, Faculty of Associated Medical Sciences, Chiang Mai University, Chiang Mai 50200, Thailand; lapamas_ru@cmu.ac.th (L.R.); natsima.v@cmu.ac.th (N.V.); yinwenxian@swmu.edu.cn (W.Y.); sawitree.chiampa@cmu.ac.th (S.C.)

[2] Cancer Research Unit of Associated Medical Sciences (AMS CRU), Faculty of Associated Medical Sciences, Chiang Mai University, Chiang Mai 50200, Thailand

[3] Department of Pharmacy, The Affiliated Hospital, Southwest Medical University, Luzhou 646000, China; liuyuanzhi121@swmu.edu.cn (Y.L.)

[4] Center of Excellence in Pharmaceutical Nanotechnology, Faculty of Pharmacy, Chiang Mai University, Chiang Mai 50200, Thailand; okng2000@gmail.com (S.O.)

[5] Department of Traditional Chinese Medicine, Faculty of Science, Chandrakasem Rajabhat University, Bangkok 10900, Thailand; methee.r@chandra.ac.th (M.R.)

[6] Department of Chemistry, Faculty of Science, Chandrakasem Rajabhat University, Bangkok 10900, Thailand; trinnakorn.k@chandra.ac.th (T.K.)

[7] Department of Pharmaceutical Sciences, Faculty of Pharmacy, Chiang Mai University, Chiang Mai 50200, Thailand

[8] Department of Chemistry, Faculty of Science, Chiang Mai University, Chiang Mai 50200, Thailand; aroonchai.s@cmu.ac.th (A.S.)

[9] Department of Applied Chemistry, Faculty of Science and Engineering, Chuo University, Tokyo 112-8551, Japan; aiwasaki686@g.chuo-u.ac.jp (A.I.)

[10] Department of Materials and Life Sciences, Faculty of Science and Technology, Sophia University, Tokyo 102-8554, Japan; chicknanga0@gmail.com (C.N.C.)

* Correspondence: t-usuki@sophia.ac.jp (T.U.); songyot.anuch@cmu.ac.th (S.A.); Tel.: +81-33238-3446 (T.U.); +66-5394-9237 (S.A.)

Content

Supplementary Table

Supplementary Table S1. IC_{50} values (µg/mL) and Selectivity index (SI) of 4 purified compounds from *A. evecta*, in KG-1a and EoL-1 leukemic cells, and PBMCs.

Supplementary Table S2. Electro-impact mass spectra (EI-MS) of purified compound 4 (Ternary mixture)

Supplementary Table S3. ^{1}H NMR of the ternary mixture.

Supplementary Table S4. ^{13}C-NMR spectra of the ternary mixture.

Supplementary Table S5. IC_{20} and IC_{50} values (µg/mL) of AE EtOAc No. 003 and the ternary mixture, isolated from AE EtOAc No. 003 in RAW264.7 macrophagic cells.

Supplementary Data

Supplementary Data S1. Infrared (IR) data of purified compound 4 (Ternary mixture)

Supplementary Figure

Supplementary Figure S1. ^{1}H-NMR spectra of the ternary mixture.

Supplementary Figure S2. ^{13}C-NMR spectra of the ternary mixture.

Supplementary Figure S3. COSY NMR of the ternary mixture.

Supplementary Figure S4. HMBC NMR of the ternary mixture.

Supplementary Figure S5. Cytotoxicity of AE EtOAc No. 003 and the ternary mixture, isolated from AE EtOAc No. 003 in RAW264.7 macrophagic cells using MTT assay.

Supplementary Table S1. IC_{50} values (μg/mL) and Selectivity index (SI) of 4 purified compounds from *A. evecta*, in KG-1a and EoL-1 leukemic cells, and PBMCs.

**Purified compounds from AE EtOAc No. 003**	**IC_{50} values (mean ± SD, μg/ml)**			**Selectivity index (SI)**	
	**KG-1a**	**EoL-1**	**PBMCs**	**KG-1a**	**EoL-1**
Purified Compound 1	>100	26.18 ± 3.65	>100	~1	3.82
Purified Compound 2	74.82 ± 5.42	16.45 ± 3.04	98.00 ± 9.56	1.31	5.96
Purified Compound 3	57.80 ± 2.54	19.86 ± 1.01	82.15 ± 6.18	1.42	4.14
Purified Compound 4 (Ternary mixture)	9.73 ± 0.24*	8.10 ± 0.73*	51.36 ± 8.37	5.28*	6.34*

Results were expressed as mean ± SD of three independent experiments

*Good cytotoxicity in each cell lines

Supplementary data of the ternary mixture

Supplementary Data S1. Infrared (IR) data of purified compound 4 (ternary mixture)

3399 cm^{-1} (O–H stretching vibration); 3065 and 3044 cm^{-1} (aromatic C–H stretching); 2918 cm^{-1} (aliphatic C–H stretching); 2329–2359 cm^{-1} (atmospheric CO_2 absorption); 1752 and 1716 cm^{-1} (C=O stretching vibration of ester, lactone, or conjugated carbonyl groups); 1690 cm^{-1} (conjugated C=O or C=C stretching); 1638 and 1603 cm^{-1} (aromatic C=C stretching vibrations); 1441, 1400, and 1363 cm^{-1} (CH_2 and CH_3 bending vibrations);1284 cm^{-1} (C–O stretching vibration); 1196 and 1159 cm^{-1} (C–O–C stretching vibrations); 1104, 1079, and 1007 cm^{-1} (C–O stretching vibrations); 948, 888, 849, 804, 773, 764, 716, and 696 cm^{-1} (aromatic C–H out-of-plane bending); 672, 618, 578, and 518 cm^{-1} (ring deformation or heavy atom vibration).

Supplementary Table S2. Electro-impact mass spectra (EI-MS) of purified compound 4 (ternary mixture)

Ternary mixture	Electro-impact mass spectra (EI-MS)
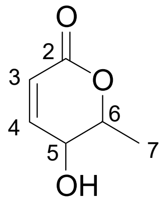 ***epi*-osmundalactone** (5,6-dihydro-5-hydroxy-6-methyl-2H-pyran-2-one)	$[M]^+$ calcd for $C_6H_8O_3$ 128.05, found 128.10
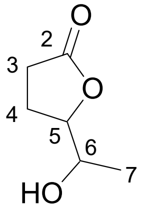 **5-(1-hydroxyethyl)-dihydro-2-furanone**	$[M]^+$ calcd for $C_6H_{10}O_3$ 130.06, found 130.10
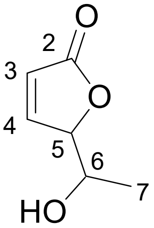 **5-(1-hydroxyethyl)-2(5H)-furanone**	$[M]^+$ calcd for $C_6H_8O_3$ 128.25, found 128.10

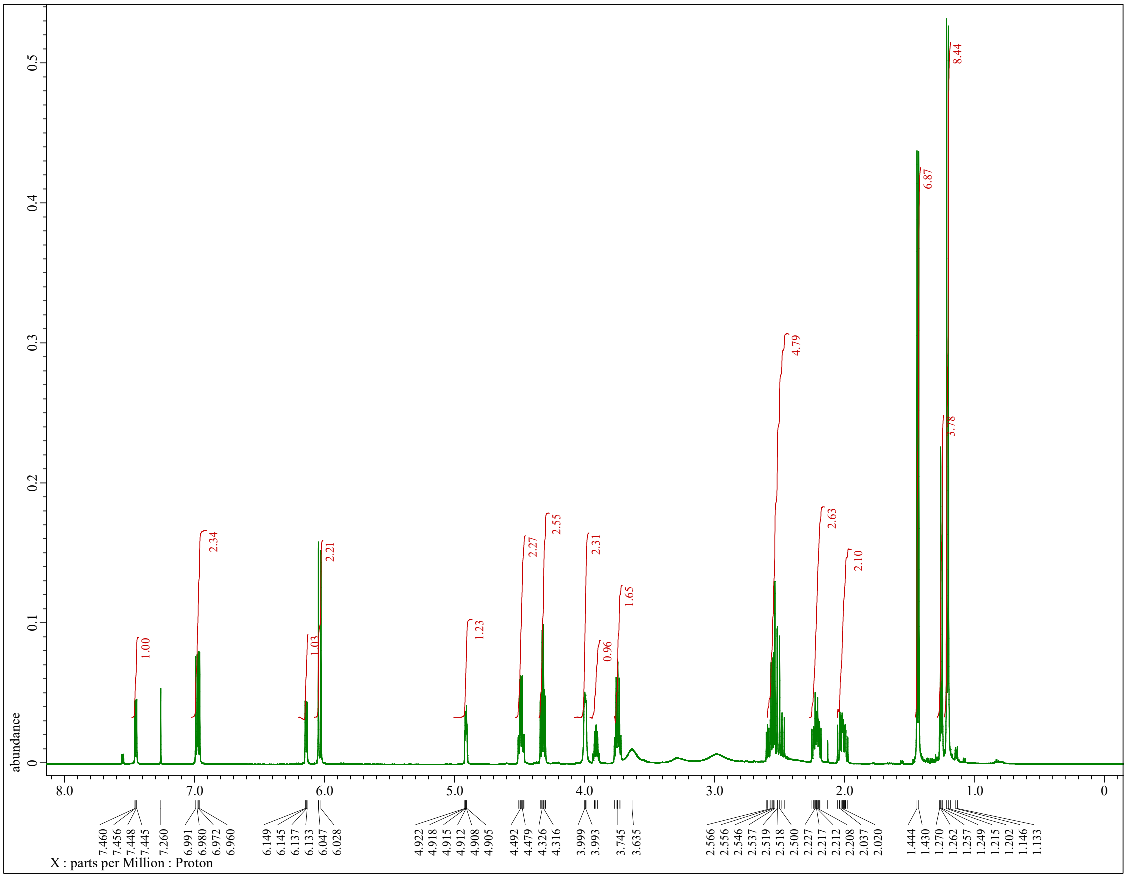

Supplementary Figure S1. ^{1}H NMR of the ternary mixture in a 2:2:1 ratio: *epi*-osmundalactone (5,6-dihydro-5-hydroxy-6-methyl-2H-pyran-2-one), 5-(1-hydroxyethyl)-dihydro-2-furanone, and 5-(1-hydroxyethyl)-2(5H)-furanone, respectively. ^{1}H NMR (500 MHz, $CDCl_3$) δ 7.45 (1H, dd, *J* = 5.8, 1.5 Hz), 6.98 (2H, dd, *J* = 9.7, 5.7 Hz), 6.14 (1H, dd, *J* = 5.9, 2.0 Hz), 6.04 (1H, d, *J* = 9.7 Hz), 4.91 (1H, td, *J* = 3.4, 1.8 Hz), 4.49 (2H, qd, *J* = 6.6, 2.8 Hz), 4.32 (2H, td, *J* = 7.4, 5.3 Hz), 4.00 (2H, dd, *J* = 5.2, 2.4 Hz), 3.91 (1H, dd, *J* = 6.3, 5.4 Hz), 3.77-3.72 (2H, m), 2.59-2.46 (4H, m), 2.25-2.18 (2H, m), 2.05-1.98 (2H, m), 1.44 (6H, d, *J* = 6.7 Hz), 1.26 (3H, dd, *J* = 6.4, 4.2 Hz), 1.21 (6H, d, *J* = 6.4 Hz)

Supplementary Table S3. ^{1}H NMR of the ternary mixture in a 2:2:1 ratio: *epi*-osmundalactone (5,6-dihydro-5-hydroxy-6-methyl-2H-pyran-2-one), 5-(1-hydroxyethyl)-dihydro-2-furanone, and 5-(1-hydroxyethyl)-2(5H)-furanone, respectively.

**Ternary mixture**	**^{1}H NMR Spectra**
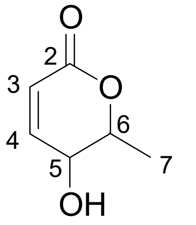 ***epi*-osmundalactone** (5,6-dihydro-5-hydroxy-6-methyl-2H-pyran-2-one)	^{1}H NMR (500 MHz, $CDCl_3$) δ 6.98 (1H, dd, *J* = 9.7, 5.7 Hz, H4), 6.04 (1H, d, *J* = 9.7 Hz, H3), 4.49 (1H, qd, *J* = 6.6, 2.8 Hz, H6), 4.00 (1H, dd, *J* = 5.2, 2.4 Hz, H5), 1.44 (3H, d, *J* = 6.7 Hz, H7)
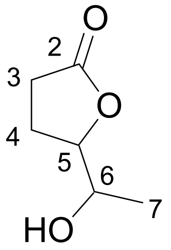 **5-(1-hydroxyethyl)-dihydro-2-furanone**	^{1}H NMR (500 MHz, $CDCl_3$) δ 4.32 (1H, td, *J* = 7.4, 5.3 Hz, H5), 3.77-3.72 (1H, m, H6), 2.59-2.46 (2H, m, H3), 2.25-2.18 (1H, m, H4), 2.05-1.98 (1H, m, H4), 1.21 (3H, d, *J* = 6.4 Hz, H7)
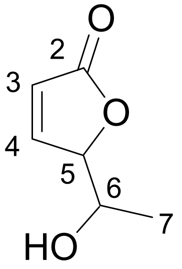 **5-(1-hydroxyethyl)-2(5H)-furanone**	^{1}H NMR (500 MHz, $CDCl_3$) δ 7.45 (1H, dd, *J* = 5.8, 1.5 Hz, H4), 6.14 (1H, dd, *J* = 5.9, 2.0 Hz, H3), 4.91 (1H, td, *J* = 3.4, 1.8 Hz, H5), 3.91 (1H, dd, *J* = 6.3, 5.4 Hz, H6), 1.26 (3H, dd, *J* = 6.4, 4.2 Hz, H7

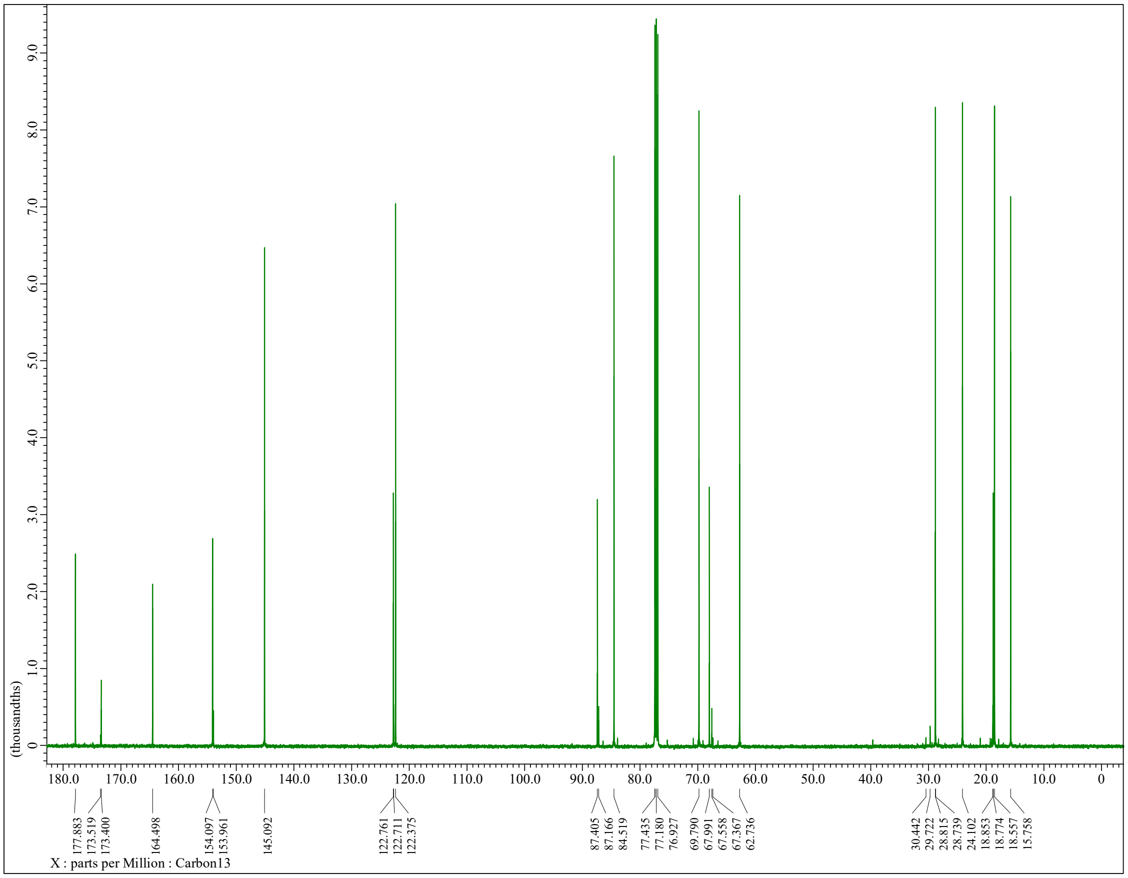

Supplementary Figure S2. ^{13}C-NMR spectra of the ternary mixture. ^{13}C-NMR (125 MHz, $CDCl_3$) δ 177.9, 173.4, 164.5, 154.1, 145.1, 122.7, 122.4, 87.4, 84.5, 77.4, 69.8, 68.0, 62.7, 28.8, 24.1, 18.8, 18.5, 15.8.

Supplementary Table S4. ^{13}C-NMR spectra of the ternary mixture

Ternary mixture	^{13}C-NMR spectra
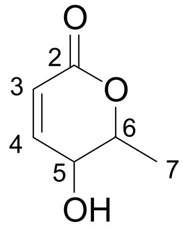 ***epi*-osmundalactone** (5,6-dihydro-5-hydroxy-6-methyl-2H-pyran-2-one)	^{13}C NMR (125 MHz, $CDCl_3$) δ 164.5(C2), 145.1(C4), 122.7(C3), 77.4(C6), 62.7(C5), 15.8(C7)
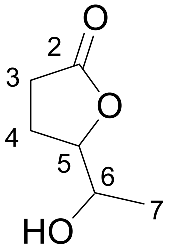 **5-(1-hydroxyethyl)-dihydro-2-furanone**	^{13}C NMR (125 MHz, $CDCl_3$) δ 177.9(C2), 84.5(C5), 69.8(C6), 28.8(C3), 24.1(C4) 18.5(C7)
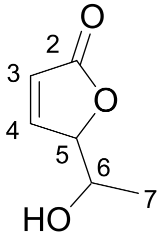 **5-(1-hydroxyethyl)-2(5H)-furanone**	^{13}C NMR (125 MHz, $CDCl_3$) δ 173.5(C2), 154.1(C4), 122.7(C3), 87.6(C5), 68.0(C6), 18.8(C7)

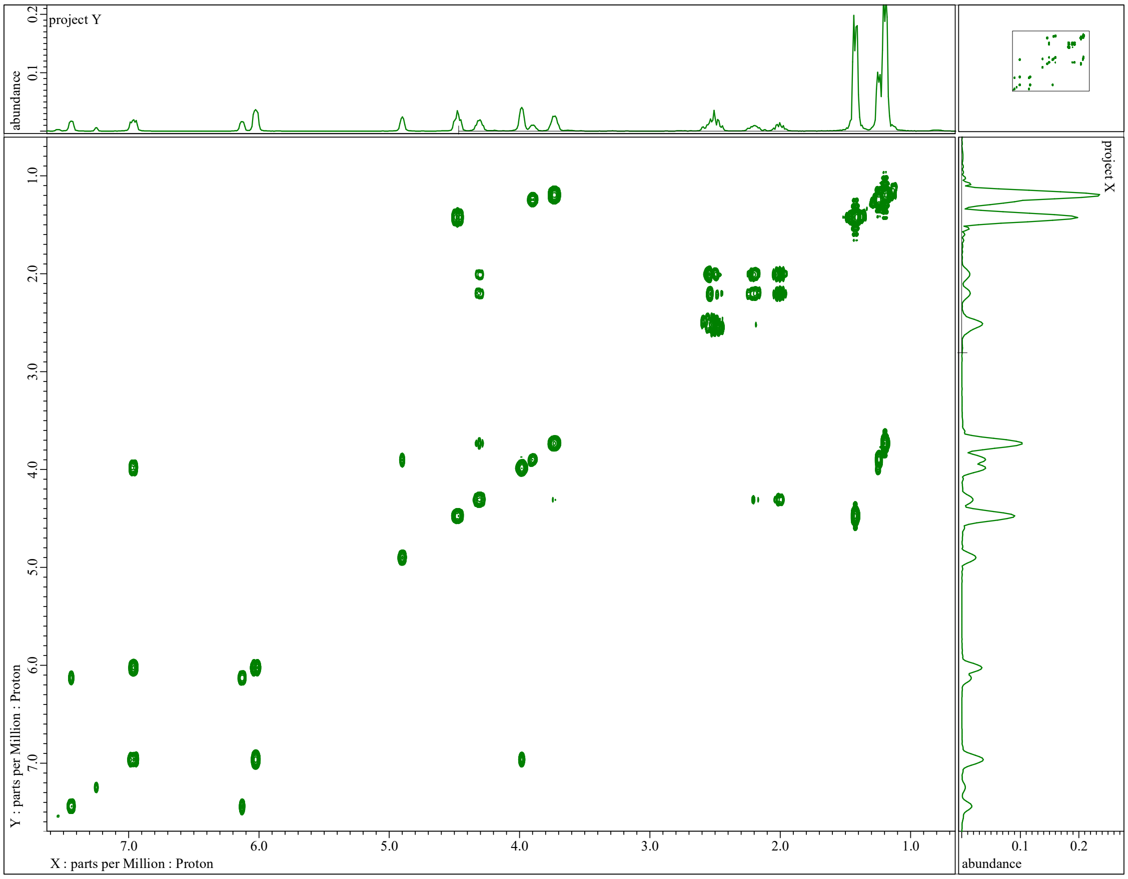

Supplementary Figure S3. COSY NMR of the ternary mixture.

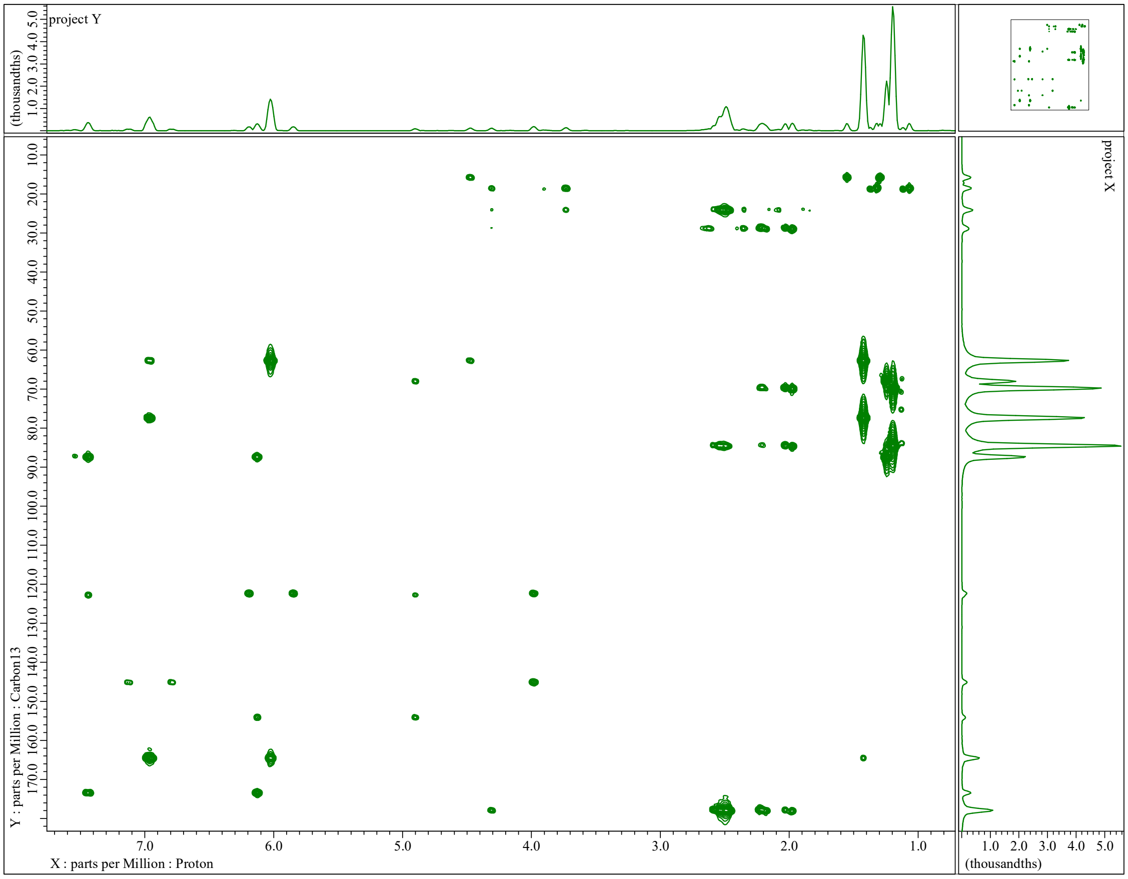

Supplementary Figure S4. HMBC NMR of the ternary mixture.

Supplementary Table S5. IC_{20} and IC_{50} values (μg/mL) of AE EtOAc No. 003 and the ternary mixture, isolated from AE EtOAc No. 003 in RAW264.7 macrophagic cells.

	**IC_{20} values (mean ± SD, μg/ml)**	**IC_{50} values (mean ± SD, μg/ml)**
AE EtOAc No.003	26.82 ± 3.40	39.81 ± 3.66
Ternary mixture	8.24 ± 0.76	10.94 ± 0.80

Results were expressed as mean ± SD of three independent experiments

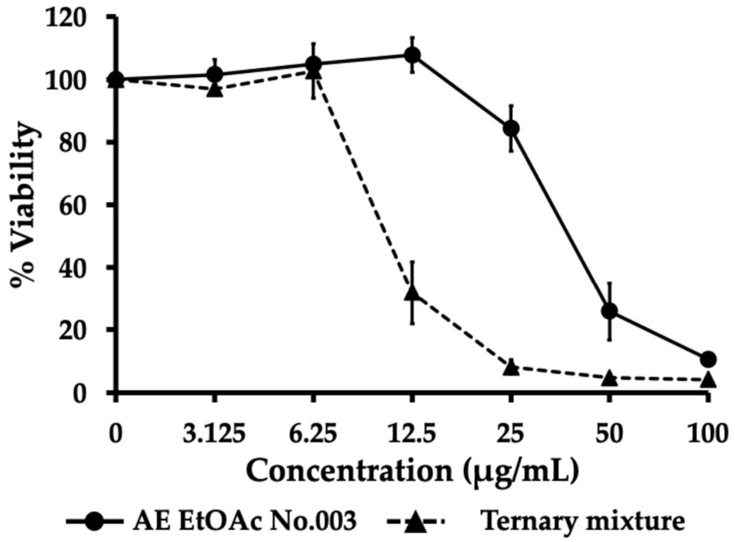

Supplementary Figure S5. Cytotoxicity of AE EtOAc No. 003 and the ternary mixture, isolated from AE EtOAc No. 003 in RAW264.7 macrophagic cells using MTT assay.
